# BCRP drives intrinsic chemoresistance in chemotherapy-naïve breast cancer brain metastasis

**DOI:** 10.1126/sciadv.abp9530

**Published:** 2023-10-18

**Authors:** Rebeca Uceda-Castro, Andreia S. Margarido, Ji-Ying Song, Mark C. de Gooijer, Hendrik A. Messal, Cecilia R. Chambers, Max Nobis, Ceren H. Çitirikkaya, Kerstin Hahn, Danielle Seinstra, David Herrmann, Paul Timpson, Pieter Wesseling, Olaf van Tellingen, Claire Vennin, Jacco van Rheenen

**Affiliations:** ^1^ Division of Molecular Pathology, Oncode Institute, The Netherlands Cancer Institute, Amsterdam, Netherlands.; ^2^ Division of Experimental Animal Pathology, The Netherlands Cancer Institute, Amsterdam, Netherlands.; ^3^ Division of Pharmacology, The Netherlands Cancer Institute, Amsterdam, Netherlands.; ^4^ Faculty of Biology, Medicine and Health, University of Manchester, Manchester, UK.; ^5^ The Christie NHS Foundation Trust, Manchester, UK.; ^6^ Cancer Ecosystems Program, Garvan Institute of Medical Research, Sydney, NSW, Australia.; ^7^ School of Clinical Medicine, Faculty of Medicine and Health, UNSW Sydney, Sydney, NSW, Australia.; ^8^ Department of Pathology, Amsterdam University Medical Centers/VUmc and Brain Tumor Center Amsterdam, Amsterdam, Netherlands.; ^9^ Princess Máxima Center for Pediatric Oncology, Utrecht, Netherlands.; ^10^ Mouse Cancer Clinic, The Netherlands Cancer Institute, Amsterdam, Netherlands.

## Abstract

Although initially successful, treatments with chemotherapy often fail because of the recurrence of chemoresistant metastases. Since these tumors develop after treatment, resistance is generally thought to occur in response to chemotherapy. However, alternative mechanisms of intrinsic chemoresistance in the chemotherapy-naïve setting may exist but remain poorly understood. Here, we study drug-naïve murine breast cancer brain metastases (BCBMs) to identify how cancer cells growing in a secondary site can acquire intrinsic chemoresistance without cytotoxic agent exposure. We demonstrate that drug-naïve murine breast cancer cells that form cancer lesions in the brain undergo vascular mimicry and concomitantly express the adenosine 5′-triphosphate–binding cassette transporter breast cancer resistance protein (BCRP), a common marker of brain endothelial cells. We reveal that expression of BCRP by the BCBM tumor cells protects them against doxorubicin and topotecan. We conclude that BCRP overexpression can cause intrinsic chemoresistance in cancer cells growing in metastatic sites without prior chemotherapy exposure.

## Teaser

Breast cancer brain metastasis cells are shown to overexpress BCRP, which protects them against doxorubicin.

## INTRODUCTION

While treatment of patients with breast cancer with chemotherapy is often initially effective, tumors regularly recur locally or systemically in a therapy-resistant form. In particular, breast cancer brain metastases (BCBMs) are often chemoresistant, which hinders the successful development of therapies for this lethal disease (*1*). The blood-brain barrier (BBB) and the blood-tumor barrier (BTB) are important contributors to extrinsic chemoresistance in BCBM because they form highly impermeable fences that block drug delivery and diffusion to the brain (*1*–*4*). The impermeability of the BBB and BTB is caused in part by brain endothelial cells that are closely connected by tight junctions, do not display fenestrations, and often express adenosine 5′-triphosphate–binding cassette (ABC) transporters that are responsible for active efflux of compounds (*2*–*5*). In addition to this extrinsic resistance, earlier exposures to drugs for the treatment of previous lesions can render cancer cells intrinsically resistant by triggering the acquisition of mutations and/or epigenetic remodeling (*6*–*8*). For example, in rodent cancer models, exposing cancer cells to cytotoxic agents can induce the up-regulation of ABC transporters that export drugs in the extracellular space, thereby rendering tumor cells intrinsically resistant (*6*–*8*).

Although both experimental and clinical data demonstrate that tumors can acquire intrinsic resistance upon exposure to chemotherapies (*7*, *9*), potential alternative mechanisms that are not triggered by drug treatment have not been characterized to the same extent. In part, this is because samples from untreated patients are scarce, particularly in patients with BCBM, rendering the study of mechanisms driving intrinsic resistance in the drug-naïve setting challenging. To overcome this, we studied drug response in primary breast tumors and BCBM that have not been exposed to chemotherapy.

We reveal that creating BCBM-like tumors using serial transplantations of chemotherapy-naïve breast tumor cells in the brain leads to the acquisition of intrinsic chemoresistance. Mechanistically, we find that chemotherapy-naïve BCBM cells are capable of undergoing vascular mimicry (VM) and simultaneously express the ABC-transporter breast cancer resistance protein (BCRP), which is commonly expressed in endothelial cells of veins and capillaries in the brain (*10*). We show that increased expression of BCRP in chemotherapy-naïve cancer cells renders BCBM cells intrinsically resistant to chemotherapies that are transported by BCRP, which can be reverted by reducing BCRP expression or activity. Together, our study identifies in the murine setting how cancer cells that grow in a foreign soil can acquire resistance to chemotherapeutics that they have never been exposed to.

## RESULTS

### BCBM is modeled in mice to study chemoresistance

To identify potential mechanisms of chemoresistance in drug-naïve brain metastases, we worked with chemotherapy naïve organoids that form brain metastasis–like lesions in the Polyomavirus middle T antigen (PyMT) (*11*) and the K14Cre, Brca1^fl/fl^, p53^fl/fl^ (KB1P) (*12*) models. Tumor organoids retain critical characteristics of the tumors from which they are derived, including slow growth kinetics, cellular heterogeneity but also low metastatic potential, and therefore faithfully model the human disease (*13*). In line with previous approaches to generating models of brain metastasis (*14*), we first injected PyMT primary organoids intracardiacally; however, mice did not develop brain tumors. We instead performed six rounds of serial intracranial transplantations of organoids isolated from primary mammary tumors derived from the PyMT and the KB1P models (*15*). From herein, breast cancer organoids enriched in the brain are referred to as BCBM. Characterization of the PyMT BCBM has previously been shown to mimic key features of the human disease in terms of pathology, immunohistochemical features, magnetic resonance imaging (MRI) and spectroscopy profiles (*15*, *16*). The KB1P BCBM model shares key features with the PyMT BCBM models, including immunohistochemical and pathological characteristics (fig. S1, A to D) (*15*). Next, we intracranially injected tumor organoids derived from primary tumors, tumors from the third or fourth round of enrichment, and the final BCBM tumors. When comparing the size of the brain tumors at 3 weeks after intracranial injection, we found that PyMT BCBM organoids had improved in vivo growth kinetics compared to organoids derived from primary tumors (fig. S1E). This difference in tumor growth correlated with increased proliferation in in vitro PyMT BCBM organoids compared to organoids derived from earlier rounds of enrichment (fig. S1F). In contrast, in the KB1P model, we did not detect significant differences in tumor size between tumors derived from BCBM organoids and primary tumor organoids (fig. S1G), and there was also no significant difference in proliferation in in vitro organoids derived from the BCBM compared to the primary tumor organoids (fig. S1H). Last, we performed bulk RNA sequencing of PyMT primary and BCBM organoids, which demonstrated an enrichment in brain-related genes in organoids derived from the BCBM compared to organoids derived from primary donor tumors (table S1). These data confirm that transcriptional selection can occur during the rounds of enrichment in the brain and is maintained in in vitro BCBM. Moreover, we previously reported that the outgrowth of BCBM organoids from intracranial injection to humane end point takes several weeks, which is an adequate time window for therapeutic testing and study of mechanisms of therapy resistance (*15*). Collectively, the generated BCBM models recapitulate key features of the human disease and can be used to study events occurring in established BCBM.

### The mouse BCBM is exposed to doxorubicin in vivo

Assessing BCBM tumor cell response to chemotherapy is challenging partly because of the impaired penetration of drugs into the brain caused by the BBB and the BTB (*17*). Chemotherapies commonly used to treat patients with breast cancer such as carboplatin are highly hydrophilic and polar and cannot permeate the lipid bilayers of the brain endothelial cells by passive diffusion (*18*). Moreover, the distribution and efficacy for less hydrophilic cytotoxic compounds including doxorubicin and topotecan are limited by the expression in the endothelial cells present in the brain of ABC efflux transporters, such as P-glycoprotein (P-gp) (also referred to as ABCB1) and BCRP.

**Fig. 1.**
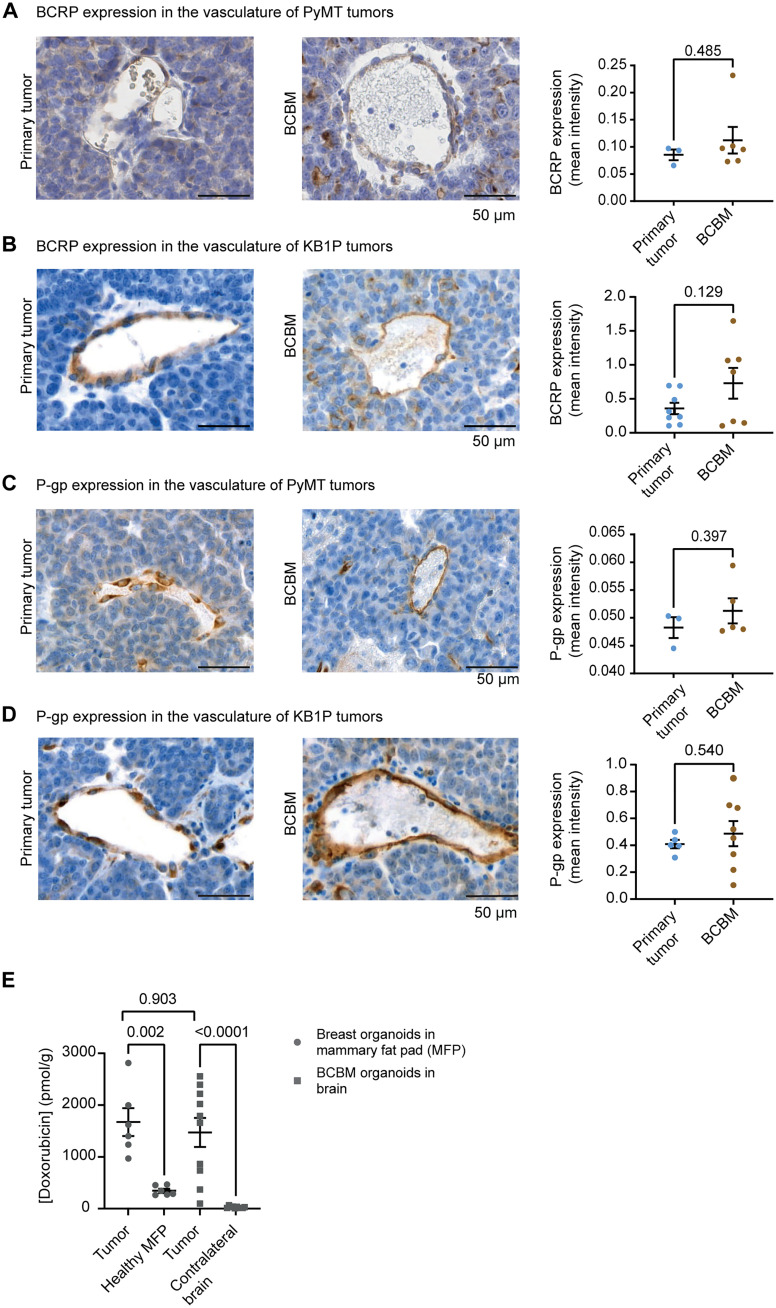
The BCBM tumor vasculature does not prevent penetration of doxorubicin. (**A**) Representative images of immunohistochemistry (IHC) of BCRP and quantification of DAB optical density (OD) in the endothelial cells forming the tumor vasculature in the PyMT model, in both primary tumors (*n* = 3 mice) and BCBM (*n* = 6 mice). (**B**) Representative images of IHC of BCRP and quantification of DAB OD in the endothelial cells forming the tumor vasculature in the KB1P model, in both primary tumors (*n* = 8 mice) and BCBM (*n* = 7 mice). (**C**) Representative images of IHC of P-gp and quantification of DAB OD in the endothelial cells forming the tumor vasculature in the PyMT model, in both primary tumors (*n* = 3 mice) and BCBM (*n* = 5 mice). (**D**) Representative images of IHC of P-gp and quantification of DAB OD in the endothelial cells forming the tumor vasculature in the KB1P model, in both primary tumors (*n* = 5 mice) and BCBM (*n* = 8 mice). (**E**) Doxorubicin concentration in breast tumors derived from PyMT primary organoids growing in the mammary gland compared to the healthy mammary fat pad and in brain tumors derived from PyMT BCBM organoids intracranially injected compared to the healthy contralateral brain hemisphere. Tissues were harvested 2 hours after treatment with doxorubicin (5 mg/kg). Data are presented as means ± SEM (for breast tumors, *n* = 6, and for brain tumors, *n* = 10).

We next assessed whether the ABC drug transporters expressed by endothelial cells (*19*–*22*) have the potential to limit the distribution and efficacy of doxorubicin in the PyMT and KB1P BCBM models, by first comparing BCRP and P-gp expression in the endothelial cells of the primary tumors versus the paired BCBMs. While we observed that endothelial cells in both sites express BCRP and P-gp, we did not find significant differences in the expression of those two ABC transporters in the endothelial cells of the BCBM versus primary breast cancer tissue in neither the PyMT nor the KB1P BCBM model (Fig. 1, A to D). To test whether the lack of enhanced expression of ABC transporters in endothelial cells correlates with diffusion of doxorubicin in the BCBM, we used chromatographic analysis to quantify the amount of doxorubicin present in the PyMT BCBM compared to the paired primary tumors. Mice bearing well-established mammary PyMT tumors or PyMT BCBM received one treatment with doxorubicin and were euthanized 2 hours after treatment. We isolated either the mammary tumor and the healthy gland or the BCBM and the contralateral brain hemisphere and measured doxorubicin concentrations in those tissues. In line with clinical data (*23*), doxorubicin concentration was higher in tumor tissues compared to healthy tissues, both in the mammary gland and in the brain (Fig. 1E). Moreover, the doxorubicin concentration is not significantly different in the primary tumor or BCBM (Fig. 1E).

Next, we used MRI with gadolinium (*24*) to confirm the leakiness of the BCBM vasculature in mice bearing PyMT BCBM (fig. S2A). In line with the detection of doxorubicin in the BCBM (Fig. 1E), we observed a stronger contrast enhancement at the BCBM periphery compared to the BCBM core, which suggests the presence of high interstitial fluid pressure (fig. S2A). Moreover, we injected mice bearing BCBM with Texas Red, a fluorescent dye with a molecular weight similar to doxorubicin (625.15 g/mol for Texas Red and 543 g/mol for doxorubicin). Using fluorescence microscopy, we observed Texas Red signal in the BCBM core and around the BCBM, indicating that the tumor vasculature is leaky (fig. S2B) (*25*). Collectively, these data demonstrate that doxorubicin penetrates our BCBM model, which warrants the study of the response to doxorubicin in chemotherapy-naïve BCBM in our model.

### Breast cancer cells enriched in the brain overexpress BCRP

**Fig. 2.**
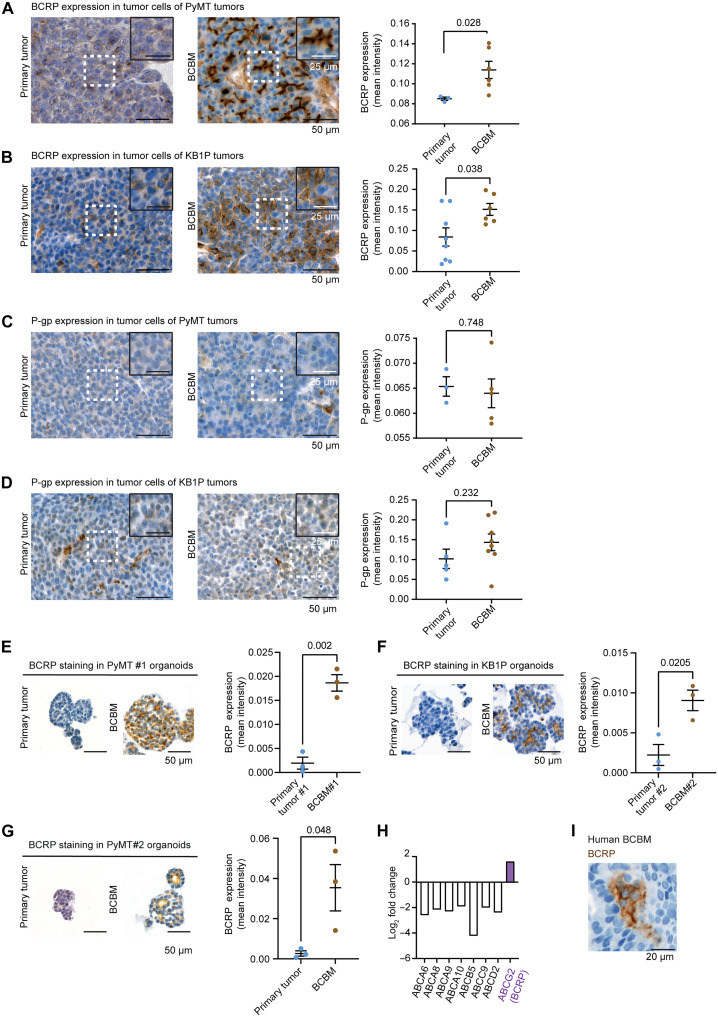
BCRP is overexpressed in breast cancer cells enriched in the brain. (**A**) Representative images of BCRP IHC staining and quantification of DAB OD in the tumor cells found in the PyMT#1 model in both primary tumors (*n* = 3 mice) and BCBM (*n* = 6 mice). (**B**) Representative images of BCRP IHC staining and quantification of DAB OD in the tumor cells found in the KB1P model in both primary tumors (*n* = 8 mice) and BCBM (*n* = 6 mice). (**C**) Representative images of P-gp IHC staining and quantification of DAB OD in the tumor cells found in the PyMT model in both primary tumors (*n* = 3 mice) and BCBM (*n* = 5 mice). (**D**) Representative images of BCRP IHC staining and quantification of DAB OD in the tumor cells found in the KB1P model in both primary tumors (*n* = 5 mice) and BCBM (*n* = 8 mice). (**E** to **G**) Representative images and quantification of DAB OD of IHC staining of BCRP in organoids derived from primary mammary tumors (left) and BCBM (right) in (E) the PyMT#1 model, (F) the KB1P model, and (G) the PyMT#2 model. For (E) to (G), *n* = 3 biological repeats with one technical replicate per repeat. For (A) to (G), data are presented as means ± SEM. (**H**) Log_2_ fold change in mRNA expression of ABC transporters genes in BCBM compared to patient-matched primary tumors from the Cosgrove dataset (*26*). (**I**) Representative images of IHC staining of BCRP in cancer cells in human BCBM samples.

While studying the expression of ABC proteins in the vasculature of the PyMT and KB1P BCBMs, we unexpectedly observed changes in BCRP expression in the tumor cells rather than in the endothelial cells (Fig. 2, A and B). Notably, BCBM cancer cells expressed significantly higher levels of BCRP compared to cancer cells of the paired primary tumors (Fig. 2, A and B), while the expression of P-gp was not significantly different (Fig. 2, C and D). Furthermore, the differential expression of BCRP was maintained in organoid cultures, suggesting that the increase in BCRP expression is stably inherited when BCBM cells are taken out of the brain environment (Fig. 2, E and F). To confirm that the enhanced expression of BCRP is the result of the serial transplantations of breast cancer cells into the brain, we generated an additional PyMT BCBM line using a second breast tumor donor (referred to as PyMT BCBM#2). Similarly to the two other lines, BCRP expression was significantly enhanced in the PyMT BCBM#2 organoids compared to the paired primary tumor organoids (Fig. 2G), further demonstrating that BCRP expression is increased in breast cancer cells upon enrichment in the brain.

We next interrogated whether BCRP^+^ cancer cells are also present in human BCBMs. We first analyzed BCRP expression in previously published RNA sequencing data obtained from human BCBM and patient-matched primary breast tumors tissues (*26*). In Her2^+^ patients, a number of ABC transporters were down-regulated in BCBM compared to their matched primary tumors; however, notably, BCRP was up-regulated in BCBM samples compared to matched primary tumors (Fig. 2H). Nonetheless, because the RNA sequencing data were obtained from whole tumor tissues, we cannot determine whether BCRP expression is increased specifically in the tumor cells, in the BTB and stroma of those BCBM samples or both. To further assess this, we performed immunohistochemistry (IHC) analysis of BCRP in samples, obtained from surgical resection in patients with BCBM. This revealed the presence of a small number of BCRP^+^ tumor cells in human BCBMs (Fig. 2I).

Last, we assessed whether the enhanced BCRP expression in metastatic cells was specific to BCBM by quantifying BCRP expression in extracranial, drug-naïve mouse metastatic tissues. We first studied primary pancreatic tumors and matched liver metastases in the mouse LoxP-Stop-LoxP (LSL)-Kras^G12D/+^;LSL-Trp53^R172H/+^;Pdx1-Cre (KPC) model (*27*). Here, we did not observe significant differences in BCRP expression in tumors generated spontaneously or by orthotopic injection (fig. S2, C and D). Moreover, we did not find a significant difference in BCRP expression in lung metastases compared to matched primary breast tumor in the keratin14Cre;Cdh1^fl/fl^;Trp53^fl/fl^ (KEP) model (fig. S2E). However, we detected a significant up-regulation of BCRP in PyMT tumor cells that spontaneously metastasized to the lungs compared to matched primary breast tumors (fig. S2F). The latter finding suggests that BCRP can also be up-regulated by tumor cells that have metastasized to other sites than the brain. However, while we consistently observe an increase in BCRP expression in multiple BCBM models, this increase does not consistently happen in extracranial metastases.

### BCRP-positive BCBM cells can undergo VM

**Fig. 3.**
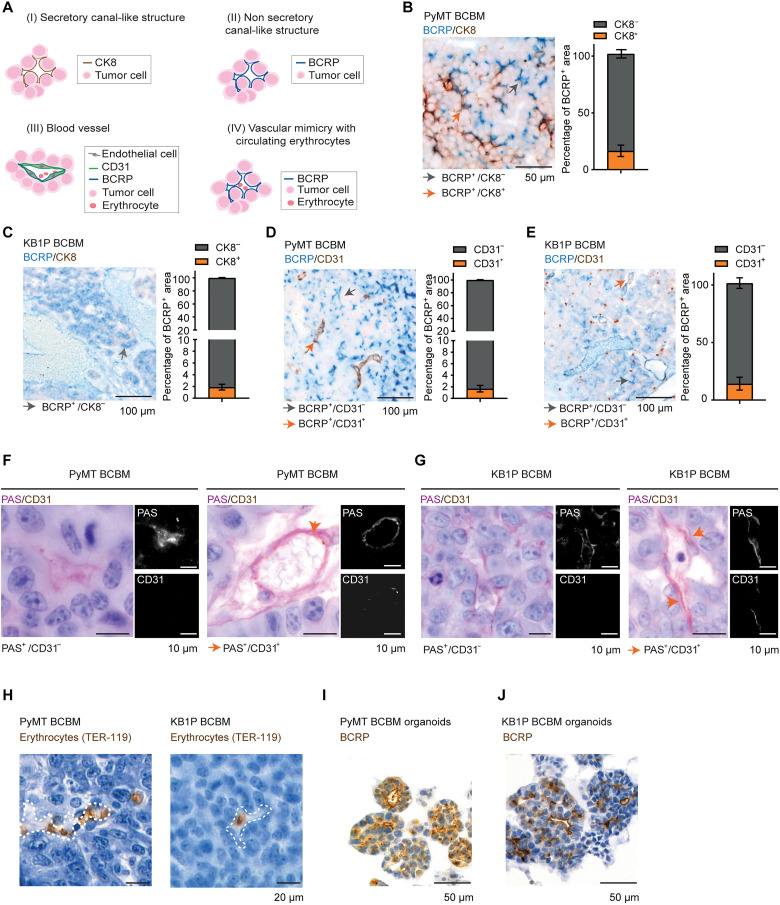
BCRP^+^ cancer cells can undergo VM in BCBM. (**A**) Schematic representation of various canal-like structures found in BCBM, and assessed in the rest of the figure via IHC. (**B** and **C**) Representative images and quantification of IHC dual staining of BCRP (blue) and CK8 (brown) in the BCBM of (B) the PyMT#1 model and (C) the KB1P model. (**D** and **E**) Dual staining and quantification of BCRP (blue) and CD31 (brown) in the BCBM of (D) the PyMT#1 model and (E) the KB1P model. Gray arrows in (B) to (E) indicate single positive staining, and orange arrows indicate double positive staining. (**F** and **G**) Representative images of IHC dual staining of PAS (pink) and CD31 (orange) in the BCBM of (F) the PyMT#1 model and (G) the KB1P model. (**H**) Representative images of IHC staining of TER-119 in the BCBM of the PyMT#1 model and of the KB1P model. Dotted lines in (H) indicate the lumen of the canal-like structures. (**I** and **J**) Representative images of IHC staining of BCRP in organoids derived from the BCBMs in (I) the PyMT#1 model and (J) the KB1P model. For this figure, data were analyzed in three mice per group.

We also observed that BCRP^+^ tumor cells formed canal-like structures in BCBM, with BCRP being polarized toward the lumen of those structures (see cartoons in Fig. 3A and images in Fig. 2, A, E, F, and G). Considering that our BCBMs originate from epithelial breast tissues, we tested whether those canal-like structures may have secretory functions by performing IHC analysis of cytokeratin 8 (CK8) expression, a marker of secretory cells [see cartoon in Fig. 3A (I)] (*28*). We observed some canal-like structures positive for CK8 [Fig. 3, B and C (orange arrows), and fig. S3A]. However, a large proportion of the BCRP^+^ cells lining the canal-like structures were negative for CK8 [Fig. 3, A (II), B, and C, and fig. S3B], suggesting that most canal-like structures lined with BCRP^+^ tumor cells do not have secretory functions. We therefore next interrogated whether the observed canal-like, nonsecretory structures displayed characteristics of a VM phenotype. VM is defined by the ability of tumor cells to transdifferentiate and to acquire features of endothelial cells [see cartoon in Fig. 3A (IV)] (*29*, *30*). VM has been hypothesized to enable the generation of a tumor-derived vasculature that sustains cancer cell growth in nonpermissive environments (*31*, *32*). In addition, VM has been reported to occur in human and mouse brain metastases and to drive tumor and metastasis formation in various sites including the brain (*33*, *34*). To characterize VM in the BCBMs, we next performed IHC analyses of CD31 (marker of endothelial cells) and histochemistry of periodic acid–Schiff (PAS). Structures that are lined with PAS and CD31^−^ cells are considered to be VM-like structures (*35*). While the vast majority of CD31^+^ cells was also BCRP^+^ (see orange arrows in Fig. 3, D and E, and fig. S3, C and D, for the images of the separate channels) a portion of canal-like structures lined with BCRP^+^ cancer cells stained negative for CD31 (gray arrows) and positive for PAS (Fig. 3, F and G). In addition, IHC of TER-119, a marker of erythrocytes (*36*), was also applied in our BCBMs. We identified a small number of erythrocytes that were present inside the canal-like structures lined with cancer cells, which is compatible with blood cells circulating through some of those canal-like structures (Fig. 3H). Last, the observed canal-like structures were also maintained in in vitro organoids (Fig. 3, I and J). These data suggest that breast cancer organoid cells that grow in the brain have the ability to form channels that are often described as VM and to concomitantly express BCRP.

### Breast cancer cells enriched in the brain become intrinsically resistant to doxorubicin

**Fig. 4.**
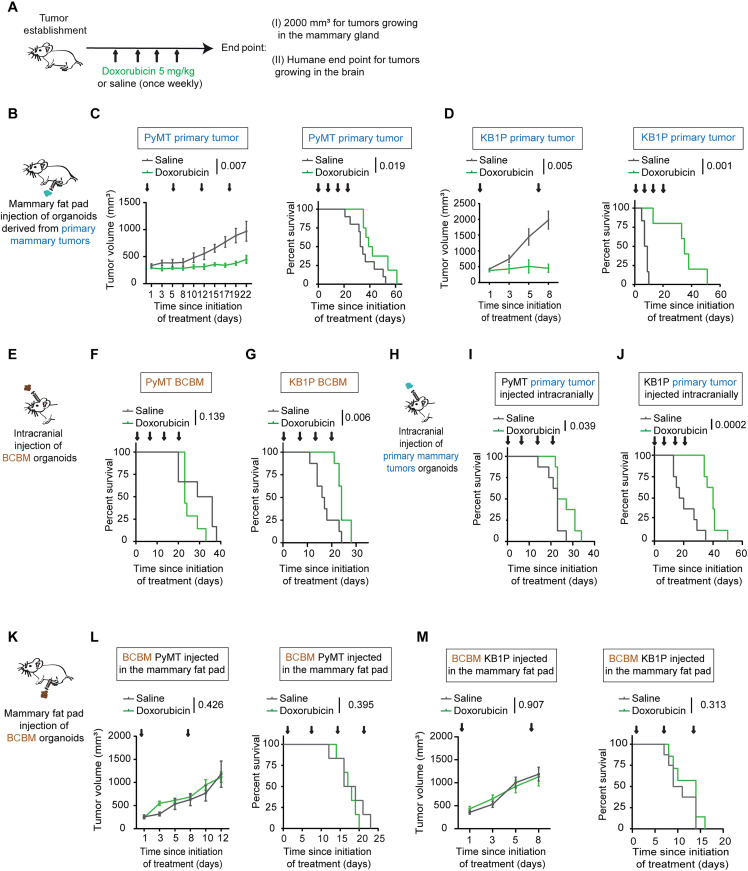
Differential sensitivity of tumors grown upon injection of primary breast and BCBM organoids. (**A**) Schematic representation of treatment timeline and (**B**) experimental setting in (C) and (D). (**C**) Tumor growth and Kaplan-Meier curves of mice bearing primary PyMT mammary tumors upon treatment with saline (*n* = 10) or doxorubicin (*n* = 7). (**D**) Tumor growth and Kaplan-Meier curves of mice bearing primary KB1P mammary tumors upon treatment with saline (*n* = 6) or with doxorubicin (*n* = 5). (**E**) Schematic representation of the experimental setting in (F) and (G). (**F** and **G**) Kaplan-Meier curves of mice bearing (F) PyMT or (G) KB1P BCBM and treated with saline or with doxorubicin. For the PyMT BCBM model, *n* = 6 mice treated with saline and *n* = 7 mice treated with doxorubicin; for the KB1P BCBM model, *n* = 8 mice per treatment group. (**H**) Schematic representation of the experimental setting in (I) and (J). (**I** and **J**) Kaplan-Meier curves of mice bearing tumors derived from (I) PyMT and (J) KB1P breast organoids injected intracranially and treated with saline (*n* = 8 mice) or with doxorubicin (*n* = 8 mice). (**K**) Schematic representation of the experimental setting of (L) and (M). (**L**) Tumor growth and Kaplan-Meier curves of mice bearing tumors generated by PyMT BCBM organoids implanted in the mammary fat pad and treated with saline (*n* = 6 mice) or with doxorubicin (*n* = 6 mice). (**M**) Tumor growth and Kaplan-Meier curves of mice bearing tumors generated by KB1P BCBM organoids implanted in the fat pad and treated with saline (*n* = 8 mice) or with doxorubicin (*n* = 8 mice). Tumor volumes plotted until the first mouse of the cohort reached the maximum tumor volume. The *P* values were calculated using a mixed-effects model with the Geisser-Greenhouse correction for growth curves and log-rank (Mantel-Cox) test for the Kaplan-Meier curves. Arrows indicate the time of intravenous administration of saline or doxorubicin.

Because BCRP can actively export doxorubicin (*37*), we next hypothesized that the enhanced expression of BCRP in the drug-naïve BCBM cancer cells may influence their response to doxorubicin. To test this, we compared the response of primary tumors and paired BCBMs to doxorubicin. Organoids derived from donor breast tumors were transplanted into the fat pad of friend leukemia virus B (FVB) mice, and organoids derived from BCBM tumors were injected intracranially. Upon tumor formation, mice bearing tumors in the mammary fat pad were randomized on the basis of tumor volume. Next, mice were subjected to weekly treatment with saline or doxorubicin (Fig. 4A). In line with previous studies (*8*, *38*), doxorubicin treatment stalled primary breast tumor growth and resulted in a prolonged survival [PyMT primary tumor, median survival of 34 days for saline-treated mice versus 40.5 days for doxorubicin-treated mice (Fig. 4, B and C); KB1P primary tumor, median survival of 8 days for saline-treated mice versus 35 days for doxorubicin-treated mice (Fig. 4, B and D)]. For mice bearing BCBM, mice were assigned to treatment groups based on in vivo imaging system (IVIS) flux signal to treat tumors of similar size (fig. S4, A and B). In contrast to the primary tumor setting, we did not observe a significant difference in survival upon doxorubicin treatment compared to saline control in mice bearing PyMT BCBM (median survival of 43 days for saline-treated mice versus 37 days for doxorubicin-treated mice; Fig. 4, E and F). Moreover, doxorubicin treatment only mildly increased the survival of mice bearing KB1P BCBMs compared to saline control (median survival of 16.5 days for saline-treated mice versus 24 days for doxorubicin-treated mice; Fig. 4, E and G). This suggests that BCBM are less responsive to doxorubicin than their primary tumor counterparts.

**Fig. 5.**
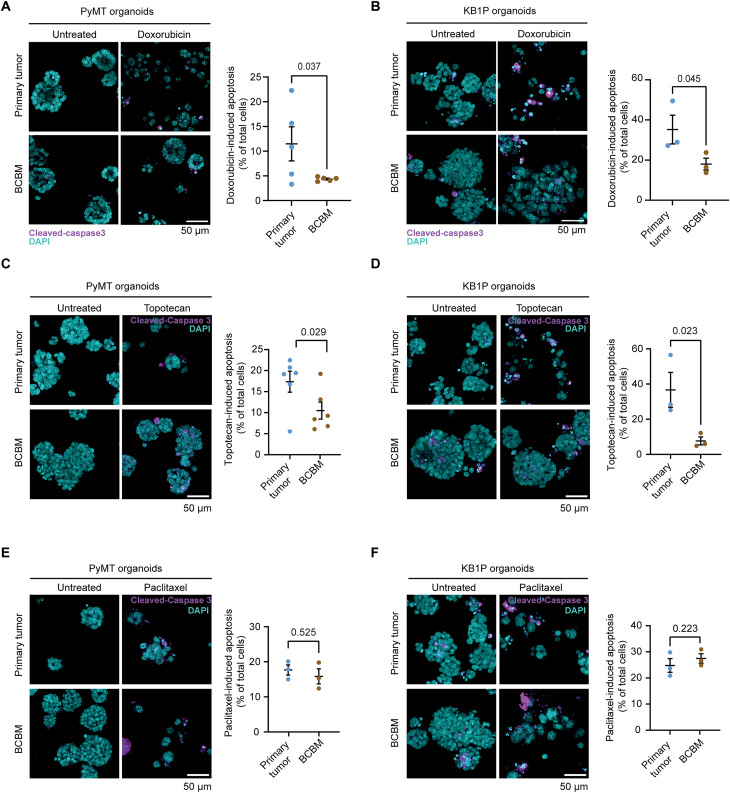
Breast cancer tumor cells enriched in the brain are intrinsically resistant to doxorubicin. (**A**) Representative images and quantification of immunofluorescent staining of cleaved caspase 3 in organoids derived from PyMT#1 primary tumors or BCBM and upon treatment with doxorubicin. *n* = 3 biological repeats with one technical replicate per repeat. (**B**) Representative images and quantification of immunofluorescent staining of cleaved caspase 3 in organoids derived from KB1P primary tumors or BCBM and upon treatment with doxorubicin. *n* = 3 biological repeats with one technical replicate per repeat. Data are presented as means ± SEM. (**C**) Representative images and quantification of cleaved caspase 3 immunofluorescent staining in PyMT#1 organoids derived from primary tumors and from BCBM and upon treatment with topotecan. (**D**) Representative images and quantification of cleaved caspase 3 immunofluorescent staining in KB1P organoids derived from primary tumors and from BCBM and upon treatment with topotecan. (**E**) Representative images and quantification of cleaved caspase 3 immunofluorescent staining in PyMT#1 organoids derived from primary tumors and from BCBM and upon treatment with paclitaxel. (**F**) Representative images and quantification of cleaved caspase 3 immunofluorescent staining in KB1P organoids derived from primary tumors and from BCBM and upon treatment with paclitaxel. *n* = 3 biological repeats with one technical replicate per repeat. Data are presented as means ± SEM.

To test whether the different response to doxorubicin was caused by the host tissue (mammary fat pad versus brain) or the type of organoid (primary tumor versus BCBM), we intracranially injected the doxorubicin-sensitive primary tumor organoids and subjected mice to the same doxorubicin-treatment schedule (Fig. 4, A and K). Mice were assigned to treatment groups based on IVIS flux signal to treat tumors of similar size (fig. S4C). In line with the data obtained at the orthotopic site (Fig. 4, C and D), doxorubicin-treatment increased survival of mice bearing brain tumors generated by primary tumor organoids in both the PyMT and the KB1P tumors (Fig. 4, I and J), albeit to a lesser extent than in the orthotropic setting (Fig. 4, C and D). Next, we performed the reverse experiment and transplanted the BCBM organoids into the fat pad of recipient mice (Fig. 4K). Upon tumor formation, mice were subjected to the same weekly treatment with saline or doxorubicin as before. In contrast to primary breast tumors (Fig. 4, C and D), neither the tumor growth nor the mouse survival was altered upon doxorubicin treatment compared to the saline treatment in any of the two models (Fig. 4, L and M). Doxorubicin intratumoral concentration was not significantly different in the tumors at the various transplantation settings (fig. S4D). Combined, these data suggest that BCBM organoids have acquired a resistance to doxorubicin that is maintained outside of the brain environment. To confirm this result, we subjected organoids derived from BCBMs and from their paired primary tumors to doxorubicin in vitro. In line with our in vivo data, doxorubicin treatment induced less apoptosis in organoids grown from BCBMs than in organoids grown from paired primary tumors (Fig. 5, A and B). Together, this demonstrates that chemotherapy-naïve cancer cells that grow in the brain have acquired an intrinsic resistance to doxorubicin.

### BCRP drives intrinsic chemoresistance in BCBM

We next asked whether the overexpression of BCRP in BCBM tumor cells drives the observed intrinsic resistance to doxorubicin. We first tested whether BCBM cancer cells specifically export BCRP substrates by subjecting organoids derived from BCBM and paired primary breast tumors to in vitro treatment with topotecan, another BCRP-specific substrate (*39*). In both paired organoid lines, topotecan induced apoptosis to a significantly lower level in the BCBM organoids compared to the primary tumor organoids (Fig. 5, C and D). In addition, treatment with paclitaxel, a compound that is not a substrate of BCRP (*40*), triggered apoptosis to a similar level in the BCBM organoids and their paired primary tumor organoids for both lines (Fig. 5, E and F). This demonstrates that chemotherapy-naïve BCBM tumor cells display a reduced response to chemotherapy drugs that are specific substrates of BCRP.

**Fig. 6.**
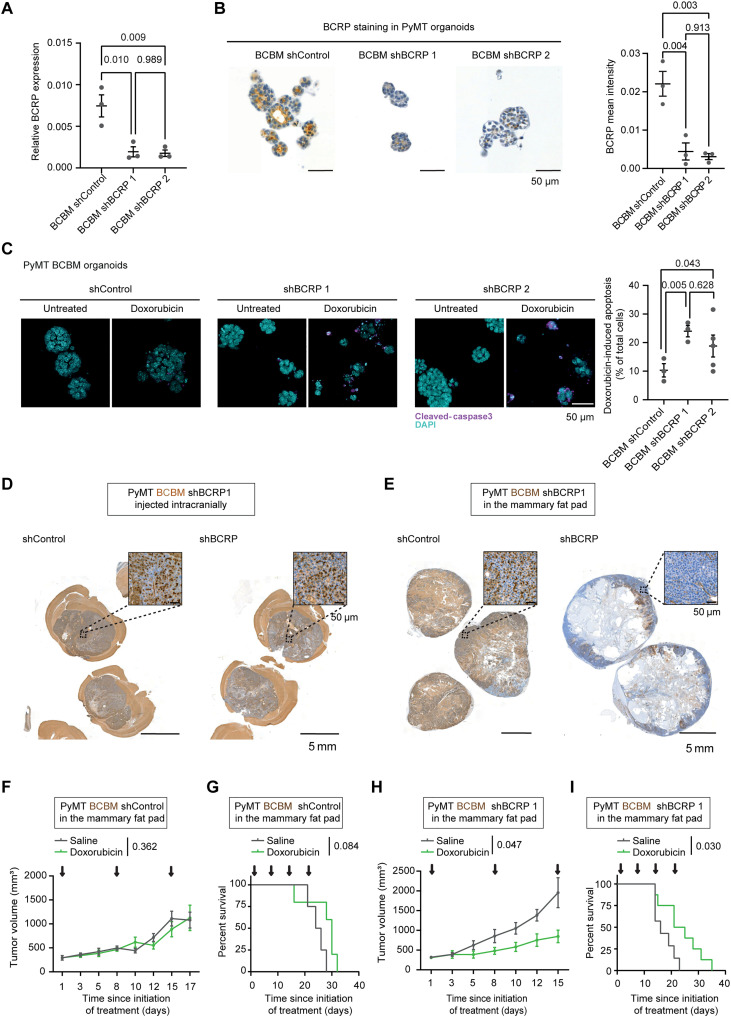
Reducing BCRP expression and activity alleviates resistance to doxorubicin. (**A**) Real-time quantitative polymerase chain reaction (qPCR) of BCRP in PyMT organoids engineered with a shRNA control (shControl) or with shRNAs against BCRP (shBCRP 1 and shBCRP 2). *n* = 3 biological repeats with three technical replicates per repeat. (**B**) Representative images and quantification of BCRP staining in organoids as indicated. (**C**) Representative images and quantification of immunofluorescent staining of cleaved caspase 3 in organoids and treatment conditions as indicated. *n* = 3 biological repeats with one technical replicate per repeat. (**D**) Representative images of BCRP IHC staining in PyMT BCBM derived from shControl and shBCRP 1 BCBM organoids intracranially injected. (**E**) Representative images of BCRP IHC staining in PyMT breast tumors derived from shControl and shBCRP 1 BCBM organoids injected in the mammary fat pad. (**F**) Tumor growth of mice bearing tumors generated by PyMT BCBM shControl organoids, transplanted in the mammary fat pad, and treated with saline (*n* = 4 mice) or with doxorubicin (*n* = 5 mice). (**G**) Kaplan-Meier curves of mice bearing tumors generated by PyMT BCBM shControl, transplanted in the mammary fat pad, and treated with saline (*n* = 4 mice) or with doxorubicin (*n* = 5 mice). (**H**) Tumor growth of mice bearing tumors generated by PyMT BCBM shBCRP1 organoids, implanted in the fat pad, and treated with saline (*n* = 7 mice) or with doxorubicin (*n* = 8 mice). (**I**) Kaplan-Meier curves of mice bearing tumors generated by PyMT BCBM shBCRP 1 organoids, transplanted in the fat pad, and treated with saline (*n* = 7 mice) or with doxorubicin (*n* = 8 mice). The tumor volumes are plotted until the first mouse of the cohort reached the maximum tumor volume. The *P* values were calculated using a mixed-effects model with the Geisser-Greenhouse correction for growth curves and log-rank (Mantel-Cox) test for the Kaplan-Meier curves.

We subsequently interrogated whether reducing BCRP expression in BCBMs would improve their response to doxorubicin. We used two short hairpin RNAs (shRNAs) and confirmed their ability to down-regulate the expression of BCRP in PyMT BCBM organoids (Fig. 6, A and B). We treated those organoids with doxorubicin or topotecan and observed a higher amount of apoptosis and a reduction in viability in BCBM organoids when BCRP expression is reduced compared to control upon treatment with doxorubicin and topotecan (Fig. 6C and fig. S5, A and B). These data show that knockdown of BCRP reduces the intrinsic resistance to doxorubicin. Next, we performed a rescue experiment by reexpressing BCRP in the PyMT BCBM organoids engineered with shRNA 1, and we confirmed that BCRP expression is enhanced in those organoids (fig. S6A and table S2 for details on the vector). We subjected those organoids to doxorubicin and found that survival after doxorubicin treatment in organoids engineered with the BCRP-shRNA1 was higher upon BCRP reexpression (fig. S6B), further confirming the specific role of BCRP in driving resistance to this compound in the BCBM organoids.

We next tested whether reducing BCRP expression affects the response to doxorubicin in PyMT BCBM cancer cells in vivo. We first intracranially injected the PyMT BCBM organoids engineered with the control-shRNA or with the BCRP–shRNA 1. In this setting, the down-regulation of BCRP expression was not maintained in tumors growing in the brain (Fig. 6D). While this observation again points to a model in which BCRP^+^ tumor cells are positively selected in this organ (Fig. 6D), the reexpression of BCRP in BCRP-shRNA1 brain tumors technically prevents us from studying the response to doxorubicin in this setting. Instead, we injected the BCBM organoids engineered with the control-shRNA and the BCRP–shRNA 1 in the mammary fat pad. Here, BCRP down-regulation was maintained in the BCRP–shRNA 1 breast tumors compared to the control-shRNA breast tumors (Fig. 6E). Upon tumor formation, mice were subjected to the same weekly treatment with doxorubicin as previously. Tumors derived from BCBM organoids engineered with the control-shRNA did not respond to doxorubicin (Fig. 6, F and G). However, the growth of tumors derived from BCBM organoids engineered with BCRP-shRNA 1 was significantly reduced upon doxorubicin treatment (Fig. 6H), resulting in an increased mouse survival compared to control-treated mice (Fig. 6I). These data show that BCRP knockdown sensitizes BCBMs to doxorubicin.

**Fig. 7.**
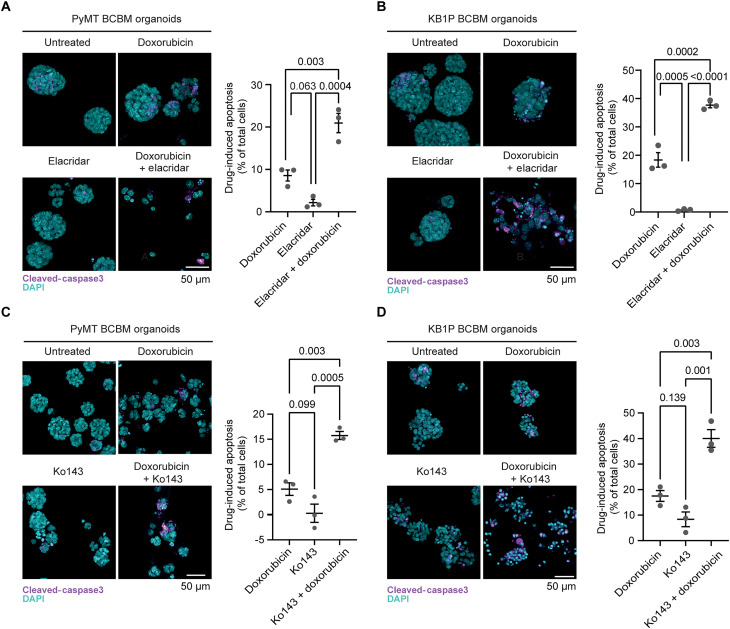
Reducing BCRP activity alleviates resistance to doxorubicin. Representative images and quantification of immunofluorescent staining of cleaved caspase 3 in organoids derived from (**A**) PyMT or (**B**) KB1P BCBM, upon treatment with dimethyl sulfoxide (DMSO), elacridar, DMSO and doxorubicin, or elacridar and doxorubicin. *n* = 3 biological repeats with one technical replicate per repeat. (**C** and **D**) Representative images and quantification of immunofluorescent staining of cleaved caspase 3 in organoids derived from (C) PyMT or (D) KB1P BCBM, upon treatment with DMSO, Ko143, DMSO and doxorubicin, or Ko143 and doxorubicin. *n* = 3 biological repeats with one technical replicate per repeat. Data are presented as means ± SEM.

Last, to further confirm the role of BCRP in driving resistance to doxorubicin, we tested whether pharmacological inhibition of BCRP can also sensitize BCBM organoids to doxorubicin. We subjected KB1P BCBM and PyMT organoids to a nonlethal dose of elacridar, a pharmacological inhibitor of BCRP (*41*, *42*), in combination with doxorubicin. In line with our findings using shRNA technology, inhibition of BCRP by elacridar increased the sensitivity of BCBM organoids to doxorubicin (Fig. 7, A and B). Because elacridar is also a potent inhibitor of P-gp, we assessed whether Ko134, a more specific inhibitor of BCRP, also affects BCBM response to doxorubicin. Combining Ko134 with doxorubicin significantly increased apoptosis in the BCBM organoids (Fig. 7, C and D). Together, these data demonstrate that targeting BCRP, either using shRNA technology or via pharmacological inhibition, alleviates intrinsic resistance in chemotherapy-naïve BCBM cancer cells.

## DISCUSSION

Chemoresistance often correlates with increased expression of ABC drug transporters that can occur upon previous exposures to chemotherapy in a range of solid tumors (*6*–*8*, *37*). However, in our experimental settings, BCBM cancer cells have never been subjected to any chemotherapy, neither in vitro nor in vivo. However, we observed increased expression of BCRP by the BCBM cells and reduced response to BCRP-specific chemotherapy drugs in both in vivo BCBM-derived and in vitro BCBM-derived organoids. Our work therefore identifies that in breast cancer mouse models, a mechanism of chemoresistance, where chemotherapy-naïve cancer cells enriched in the brain, can undergo VM and concomitantly overexpress BCRP, which protects them against chemotherapy. We also noted that doxorubicin concentrations in tumors derived from primary organoids were not significantly higher than in tumors grown from BCBM organoids. This observation may be explained by the fact that total tumor concentrations were measured, which does not distinguish intracellular versus extracellular concentrations. It is also possible that doxorubicin is sequestered in the BCBM tumors in nonfunctional VM-like channels or inside intracellular compartments, which would explain why the drug not only is not clear but also does not induce tumor cell death.

The brain environment is an important source of selective pressure for tumor cells metastasizing in this unique site (*1*, *43*). Previous work has demonstrated that tumor cells growing in the brain can adapt by acquiring “brain-like” properties to efficiently form metastatic masses in the brain (*44*). In line with this, we demonstrate that breast cancer cells enriched in the brain form channel structures. On the basis of their morphology and on the expression of particular markers, these structures qualify as what has been described in the literature as VM. Moreover, cancer cells lining those VM-like channels express BCRP, an ABC transporter that is classically expressed by endothelial cells in capillaries and veins of most organs including the brain (*39*, *45*, *46*). It is possible that transitioning toward a brain endothelial state enables tumor cells to survive and grow in the brain. Alternatively, the presence of BCRP expressing tumor cells may indicate differentiation toward lactating ductal cells (*47*); however, the channel-like structures that we characterize do not resemble lactating ducts morphologically. Regardless of the differentiation route, transitioning toward channel-forming cells with high BCRP expression appears to enable tumor cells to grow in the brain. Although BCRP expression was much lower in cancer cells in the primary tumors compared to cancer cells in the BCBM, we could occasionally find some BCRP^+^ cancer cells in the mouse mammary primary tumors. The inability to maintain low BCRP expression when BCBM organoids engineered with an shRNA against BCRP are injected in the brain also points to a model in which BCRP^+^ cells are positively selected for colonizing the brain. However, further work is required to confirm causality. However, our study implies that by adapting to a secondary environment such as the brain, metastatic breast tumor cells can become resistant to chemotherapy in a setting where they have never been subjected to these compounds.

Previous studies have suggested that while ABC transporters may mediate chemoresistance in murine tumors, this may be less relevant in the human setting (*48*). However, most of those studies have focused on the role of P-gp, not BCRP, and were conducted without specific selection of patients whose tumors had high expression of ABC transporters (*49*). In the murine setting, we find that the chemoresistance of chemotherapy-naïve murine BCBM is mediated by expression of the ABC transporter BCRP. In addition, BCRP also appears to be up-regulated in human BCBM (Fig. 2H). However, the vast majority of patients with BCBM has been treated with multiple chemotherapies at diagnosis (*50*–*53*); consequently, we cannot confirm in patient samples whether BCRP is also up-regulated when chemotherapy-naïve human cancer cells colonize the brain. However, a handful of studies in clinical cohorts have linked the expression of a number of ABC transporters with increased metastatic potential. For instance, ABCB1 expression has been linked with increased metastasis in prostate cancer, uveal melanoma, and breast cancer patient cohorts, while the expressions of ABCB5 and of ABCC1 were up-regulated in metastases of patients and patients with melanoma, respectively (*54*–*57*). Although those studies may point to a broad role of ABC transporters in driving metastasis, the data emanating from those studies were not performed in the drug naïve setting, in contrast to our work.

Here, we have revealed in the murine setting how cancer cells that colonize a metastatic site can adopt traits that make them chemoresistant without being exposed to therapies. Our data warrant further investigation of whether chemotherapy-naïve breast cancer cells may follow similar routes to chemoresistance in the human setting, potentially by up-regulating non-ABC transporters that are also expressed by endothelial cells of the BBB, such as solute carrier transporters (*58*). These efforts may provide a better understanding of drug resistance in BCBM to, in turn, help developing a better cure for this disease in the future. In light of efforts to develop approaches to shuttle chemotherapeutics such as doxorubicin over the BBB and BTB (for instance, in clinical trials NCT01818713, NCT03387917, or NCT02536183), the intrinsic resistance to these compounds forms a second barrier that needs to be overcome for successful therapeutic outcomes. Encouragingly, a number of studies using positron emission tomography tracers have assessed the potency of ABC transporter inhibitors to improve drug delivery to the brain (*59*, *60*). Our data suggest that targeting ABC transporters in the tumor cells in addition to targeting the BBB and BTB may be beneficial to improve responses to chemotherapy.

In conclusion, our work illustrates how murine cells that adapt to a new environment can acquire resistance to a therapy that they have never been exposed to. Future research will determine whether this or a similar mechanism may apply in the human setting.

## MATERIALS AND METHODS

### Organoid culture

Organoids were generated from end-stage, fully established mammary tumors derived from =PyMT (*11*) or KB1P (*12*) female mice. Organoids were cultured in 50-μl drops of Cultrex PathClear Reduced Growth Factor Basement Membrane Extract Type 2 (BME; Amsbio, catalog no. 3533-005-02) in either Dulbecco’s modified Eagle’s medium (DMEM)/F12 GlutaMAX (Gibco, catalog no. 10565018) supplemented with 10 mM Hepes (Gibco, catalog no. 15630106), streptomycin (100 g/ml), penicillin (100 U/ml; Gibco, catalog no. 15140122), 2% B27 (Thermo Fisher Scientific, catalog no. 17504-044), and fibroblast growth factor (FGF; 12 ng/ml; Invitrogen, catalog no. PHG0261) for organoids derived from the PyMT model; or in advanced DMEM/F12 (Thermo Fisher Scientific, catalog no. 12634-010) containing 10 mM Hepes (Thermo Fisher Scientific, catalog no. 15630-056), penicillin/streptomycin (10,000 U/ml; Thermo Fisher Scientific, catalog no. 15140-122), 2% B27 (Thermo Fisher Scientific, catalog no. 17504-044), 1.25 mM *N*-acetylcysteine (Sigma-Aldrich, catalog no. A9165), and FGF (12 ng/ml; Invitrogen, catalog no. PHG0261) for organoids derived from the KB1P model. Organoids were cultured in 20% O_2_ and 5% CO_2_ at 37°C. The absence of mycoplasma was routinely confirmed in organoids cultures using the MycoAlert PLUS kit (Lonza, catalog no. LT07-118). Organoids were passaged using TrypLE Express (Gibco, catalog no. 12605010) while shaking at 900 rpm for 10 to 15 min at 37°C.

### Mice

Animal experiments described in this study were run in accordance with the Dutch national guidelines for animal experiments and the Australian code of practice for the care and use of animals for scientific purposes and were approved by the Animal Welfare Committee of the Netherlands Cancer Institute and the Garvan Institute/St. Vincent’s Hospital Animal Ethics Committee, respectively. Animals were housed at the Netherlands Cancer Institute facility in Amsterdam, The Netherlands. Mice had access to chow and water ad libitum and were kept under specific pathogen–free conditions in individually ventilated cage. All mice used in this study were FVB female mice, aged 8 to 20 weeks old at the time of intracranial or fat pad injections. Mice were purchased from Janvier and were allowed to acclimatize for 1 week upon arrival before initiating the experiments.

### Transplantation of tumor organoids or tumor pieces in the mammary fat pad and monitoring of tumor growth

Twenty-four hours before surgery, Rimadyl (0.067 mg/ml; Zoetis) in the drinking water was administered to mice and was maintained for 72 hours following surgery. Immediately before surgery, mice were sedated with 2% (v/v) isoflurane, which was maintained throughout the course of the surgery. The skin above the right fourth mammary gland was shaved and disinfected using betadine. The fat pad was exposed by making a small incision below the nipple. A total of 100,000 single cells derived from KB1P organoids or from PyMT organoids were resuspended in 30 μl of BME (Amsbio, catalog no. 3533-005-02) and injected into the fat pad using a 30-gauge insulin syringe. The syringe was kept inside the fat pad for 30 s to avoid cells leaking out of the fat pad. Next, the skin was sutured, and mice were allowed to recover on a heating pad. Following transplantation of tumor organoids, mice were weighed and monitored, and tumor volume was measured with calipers three times per week until reaching experimental end point. The researcher performing the tumor volume measurements was blinded to the treatment group. Experimental end point was reached when tumor volume reached 2000 mm^3^.

### Intracranial injection and monitoring of tumor progression

Twenty-four hours before intracranial injection, mice were administered with Rimadyl (0.067 mg/ml; Zoetis) in the drinking water, which was maintained for 3 days following surgery. Mice were also treated with temgesic (0.1 mg/kg; Indivior Europe Limited) via subcutaneous injection 30 min before and 24 hours following surgery. Mice were sedated with 2% (v/v) isoflurane via inhalation, and their eyes were covered with duratears (Alcon). The head of the mouse was shaved and disinfected with betadine before being fixed on a stereotactic apparatus. The periosteum was revealed by making an incision in the skin and was subsequently dissected away to expose the bregma. Lidocaine (1 mg/ml; Fresenius Kabi) and bupivacaine (0.25 mg/ml; Actavis, Aurobindo Pharma B.V.) diluted in NaCl were applied to the skull as a local anesthetic. The bregma was used as a 0 reference point to determine the position for intracranial injection: 1.5 mm to the right and 1.5 mm caudal of the bregma. At this coordinate, a sterile compact drill bit was used to drill a hole in the skull. Next, a 10-μl glass Hamilton syringe with a 30-gauge and point-4 style needle was used to inject the tumor cell suspension, at a depth of 1.5 mm. A total of 40,000 single PyMT BCBM cells or 120,000 single KB1P BCBM cells were injected in 2 μl of organoid medium. Before retracting the syringe, a 2-min waiting time was given to avoid cells leaking out of the injection site. The skin around the injection site was sutured, and mice were allowed to recover on a heating pad. Mice were closely monitored during the days following surgery. Mouse weight and behavior were monitored three times a week after intracranial injection and during treatment. To follow BCBM growth and for later analysis, BCBM organoids were engineered to express an H2B-Dendra2-luciferase construct. When mice demonstrated signs of sickness due to BCBM burden (weight loss, bump on the head, loss of reflexes, and apathy), mice were monitored weekly. Experimental end point was reached when mice stopped eating or drinking, lost more than 15% of their body weight within 2 days, lost more than 20% of their body weight since the initiation of the experiment, had severe circulation or breathing problems, or had severe aberrant behavior/movement. The researcher performing the monitoring of the mice and determining experimental end points was blinded to the treatment group.

### Serial intracranial transplantation of breast cancer cells

End-stage primary breast tumors isolated from PyMT or KB1P mice were harvested and frozen in recovery cell culture freezing medium (Gibco, catalog no. 12648-010). For the PyMT model, before the first enrichment round in the brain, frozen PyMT tumor pieces from breast tumors were thawed and digested. For the KB1P model, organoids derived from primary KB1P tumors were made as single cells using TripLE. Single cells were resuspended in 3 μl of phosphate-buffered saline (PBS) and intracranially injected in recipient mice (enrichment round no. 1). Upon tumor formation in the brain of the recipient mice, the brain tumor was isolated, cut into smaller pieces, and frozen in recovery cell culture freezing medium (Gibco, catalog no. 12648-010) before being digested again and injected intracranially into a second batch of recipient mice (enrichment round no. 2). A total of six serial rounds of intracranial transplantations were performed to enrich tumor cells in the brain. BCBM organoids described in this study were isolated from BCBMs after the sixth round of enrichment.

### In vivo treatment

For mice bearing primary tumors, mice were randomized on the basis of tumor volume, with a tumor volume ranging from 200 to 400 mm^3^ and equal averaged volume per treatment group. For mice bearing BCBM, tumor growth was monitored via bioluminescence imaging on an IVIS twice per week. Mice were assigned to treatment groups based on IVIS total flux value. Both for primary tumors and BCBM, mice were treated as follows: saline vehicle (control for doxorubicin): Mice were treated every 7 days, with a maximum of four treatments with saline (same volume as doxorubicin), administered via intravenous injection; doxorubicin treatment: Mice were treated every 7 days, with a maximum of four treatments with doxorubicin (5 mg/kg; Actavis), administered via intravenous injection.

### Magnetic resonance imaging

Mice bearing BCBM were imaged using MRI 2 weeks following intracranial injection of BCBM organoids. This corresponds to the initiation of the saline or doxorubicin treatment in vivo. MRI was performed with a 7-T BioSpec 70/20 USR (Bruker, Billerica, MA USA). T1-weighted postcontrast sequence with a 3-ms echo time, 235-ms repetition time, and a flip angle of 30° was used. Gadoterate meglumine (Dotarem; 0.025 mmol/ml; Guerbet, Villepinte, France) was used as a contrast agent and was injected intravenously through a cannula inserted into the tail vein. Mice were sedated with 2% (v/v) isoflurane before and during imaging, and their heart rate and frequency were monitored throughout the procedure. Image acquisition and processing were performed with Paravision software (v6.0.1; Bruker).

### Texas Red analysis

Following MRI, mice bearing BCBM were administered with Texas Red (6 mg/kg; sulforhodamine 101, Invitrogen, catalog no. S359). Thirty minutes later, mice were anesthetized with isoflurane and perfused with saline. The brain was next isolated and frozen on dry ice using Tissue-Tek (Sakura Finetek Europe BV, Alphen aan den Rijn, The Netherlands). Subsequently, the brains were sliced and imaged using an Axio Scan.Z1 (Carl Zeiss, Oberkochen, Germany). To distinguish the BCBM and the healthy brain tissue, BCBM were engineered to express H2B-Dendra2-luciferase. The intensity of Texas Red signal and the area covered by H2B-Dendra2^+^ BCBM cells were measured in ZEISS ZEN (blue edition) software.

### Measurements of doxorubicin concentration in tumor and healthy tissues

Two hours after administration, animals were euthanized, and the breast tumor, contralateral mammary fat pad, brain tumor, and contralateral hemisphere were collected and weighted. Brain, mammary fat pad, and tumor samples were homogenized in 1% (w/v) bovine serum albumin using a FastPrep-24 (MP Biomedicals, NY). Next, 50 μl of homogenate was vortex-mixed with 5 μl of daunorubicin [1 μM in dimethyl sulfoxide (DMSO); Internal Standard] and 300 μl of ice-cold acetonitrile:fomic acid (100 + 1; v/v). Following 10 min at −20°C, the samples were centrifuged (20,000*g* for 5 min at 4°C), and 100 μl of supernatant was mixed with 400 μl of water. Next, 10 μl was injected into a liquid chromatography–tandem mass spectrometry system that consisted of an UltiMate 3000 Autosampler and high-performance liquid chromatography pump (Thermo Fisher Scientific, Waltham, MA, USA) and API3500 tandem mass spectrometry (SCIEX, Framingham, MA, USA). Separation was performed on a Zorbax Extend C18 column (2.1 mm × 100 mm particle size, 3.5 μM; Agilent, Santa Clara, USA) preceded by a SecurityGuard C18 precolumn (Phenomenex, Utrecht, The Netherlands). Mobile phase A (0.1% formic acid in water) and phase B (methanol) were used in a 5-min gradient from 20 to 95% phase B maintained for 3 min, followed by re-equilibration at 20% phase B. Multiple reactions monitoring (MRMs) for acquisition were 544.0/397.0 (doxorubicin) and 528.1/321.1 (daunorubicin).

### Study of BCRP expression in extracranial metastases

#### KPC genetically engineered mouse model

Primary pancreatic tumors and liver metastases were isolated from KPC mice. KPC mice were bred at the Australian BioResources, and at the time of weaning, tail samples were obtained for genotyping by the Garvan Molecular Genetics facility. Mice with the appropriate genotypes were ordered at ~6 weeks of age, and weighed, palpated, and monitored once weekly until detection of a palpable tumor, after which mice were weighed, palpated, and monitored 3× weekly. Mice were euthanized upon reaching study end point that included weight loss of ≥20% compared to the maximum body weight measured over the course of the study or overnight weight loss of ≥10%; tumor interferes with mobility and affects access to food and water, determination of a body condition score (BCS) of ≤2, gross abdominal distension indicating development of ascites, prolonged diarrhea for ≥3 days, and signs of systemic illness. Mice were removed from the study if they had to be euthanized because of unspecific end points not related to pancreatic cancer, including a severe prolapse diameter of ≥7 mm, a protrusion of ≥4 mm, ulcerations, dry tissue, dark red to black color, and a papilloma size of ≥10 × 10 mm, that cannot be removed surgically, signs of lymphomas in the neck or flank. Pancreatic tumors and liver metastases were isolated at humane end point.

#### KPC orthotopic model

This model was previously described here (*61*). Briefly, 50 cancer cells and 150 cancer-associated fibroblasts isolated from end-stage KPC tumors were coinjected into the pancreas of NOD/SCID/IL2Rγ (nonobese diabetic/severe combined immunodeficient/interleukin-2 receptor γ) mice during open laparotomy. Mice were euthanized at humane end point, and pancreatic tumors and liver metastases were isolated.

#### KEP model

The KEP metastasis model has been applied as previously described (*62*). Briefly, mammary tumor pieces of 1-mm^2^ size derived from spontaneously developed tumors in KEP mice were orthotopically transplanted into the mammary fat pad of 9-week-old wild-type recipient FVB mice. Mammary tumors were surgically removed when they reached the size of 100 mm^2^. Mice were intraperitoneally injected twice weekly with 100 μg of rat immunoglobulin G2a (clone 2A3, BioXCell) starting 14 days after mastectomy, when all mice have established metastases in the lung and/or lymph node, and treatments continued until the experimental end point. Mice were euthanized when they developed signs of distress caused by metastatic disease (respiratory distress) or when lymph node metastasis reached the size of 225 mm^2^.

#### PyMT lung metastasis model

A total of 1 × 10^6^ PyMT cells were injected in the mammary fat pad in NOD.Cg-Prkdcscid Il2rgtm1Wjl/SzJAusb female mice, aged 6 weeks. Mice were euthanized 5 months after transplantation, and mammary tumors and lung tissues were isolated and processed for IHC.

### Histochemistry and IHC

#### In vivo samples

Tumors grown in the mammary fat pad, brain, and lungs were collected and fixed in EAF (ethanol/acetic acid/formaldehyde/saline at 40:5:10:45, v/v), and the tumors grown in the pancreas and liver were fixed in 10% buffered formalin before being embedded in paraffin. Sections were stained with hematoxylin and eosin and PAS according to standard procedures. For IHC, 4-μm-thick sections were stained with BCRP (Cell Signaling Technology, catalog no. 42078S; 1:300), multidrug resistance 1 (MDR-1/ P-gp) (Cell Signaling Technology, catalog no. 13978; 1:200), CD31 (Abcam, catalog no. 28364; 1:500), CK8 (DSHB University of Iowa, Troma I; 1:100), TER-119 (BD Biosciences, catalog no. 550565; 1:1000), glial fibrillary acidic protein (BioTrend, catalog no. BT46-5002-04; 1:500), and E-cadherin (Cell Signaling Technology, catalog no. 3195; 1:100) antibodies. Slides were scanned with a Panoramic P1000 slide scanner (Leica Biosystems) and reviewed with Slide Score (Slide Score B.V.). Staining intensity was quantified using QuPath 0.2.1 (GitHub). A classifier was built to detect cancer cells and endothelial cells in the entire sample section. Per section and to account for the heterogeneity of BCRP and P-gp expression, more than 100,000 cells were analyzed. The averaged mean 3,3′-Diaminobenzidine (DAB) optical density measured in the cell membrane and cytoplasm is depicted in the figures of this manuscript.

Quantification of the dual staining was performed in Fiji/ImageJ software. First, a mask was applied with a color deconvolution, and the resulting images were converted to 8-bit files. Next, the areas stained only for BCRP and only for CD31/CK8 were quantified. We then created a mask based on BCRP expression and used it to quantify the area stained for both BCRP and CD31/CK8. Last, the percentage of BCRP^+^ area stained for BCRP only and for both BCRP and CD31/CK8 was calculated.

#### In vitro samples

Organoids were collected and washed in medium and PBS to remove the BME. Next, organoids were fixed in 4% paraformaldehyde for 20 min. Organoids were washed in PBS twice before being embedded in 4% agarose. Once the agarose had solidified, organoids in agarose were moved to formalin and embedded in paraffin. Four-micrometer-thick sections were stained with BCRP (Cell Signaling Technology, catalog no. 42078S; 1:300) antibody. Slides were scanned with a Panoramic P1000 slide scanner (Leica Biosystems) and reviewed with Slide Score. The intensity of BCRP staining was quantified in Fiji as described before. Briefly, color deconvolution was applied on H DAB images, and the same threshold was applied to all images to remove background signal. The mean gray values were quantified using the “analyze particle option,” normalized to the number of cells per field of view, and averaged for each biological repeat (*63*).

#### Human samples

All the human BCBM biospecimens are obtained at the Amsterdam University Medical Centers (UMC) [location Vrije universiteit Medisch Centrum (VuMC)] and have been executed pursuant to the ethical rules and regulations of the Amsterdam UMC (location VuMC). Hence, the procedures comply both with (inter)national legislative and ethical standards. Patients at Amsterdam UMC (location VuMC) were informed before brain tumor surgeries and provided the opportunity to decline the use of their biospecimens. For all samples in our study, patients did not raise objections, implying their consent for biospecimen use. In the database of the Amsterdam UMC (location VuMC), eight samples of brain resections with a metastasis of mammary carcinoma were selected. These were all invasive ductal carcinomas, not otherwise specified, with a different receptor status: two endoplasmic reticulum–positive, two HER2-positive, two triple negative, and one unknown receptor status. IHC of the formalin-fixed paraffin-embedded (FFPE) tumor samples was performed on a BenchMark Ultra autostainer (Ventana Medical Systems). Briefly, paraffin sections were cut at 3 μm, heated at 75°C for 28 min, and deparaffinized in the instrument with EZ prep solution (Ventana Medical Systems). Heat-induced antigen retrieval was carried out using Cell Conditioning 1 (Ventana Medical Systems) for 64 min at 95°C.

BCRP/ABCG2 was detected using clone D5V2K (1:100 dilution, 36 min at room temperature; Cell Signaling Technology). Bound antibody was detected using the OptiView DAB Detection Kit (Ventana Medical Systems). Slides were counterstained with Hematoxylin and Bluing Reagent (Ventana Medical Systems). A PANNORAMIC 1000 scanner from 3DHISTECH was used to scan the slides at a ×40 magnification.

### In vitro treatments

Organoids were platted as small organoids (4 to 10 cells) and treated for 72 hours with chemotherapy. The PyMT breast tumor and BCBM tumor organoids were treated with 30 nM doxorubicin (Actavis), 50 nM topotecan (Sandoz), 50 nM paclitaxel (Fresenius Kabi), 200 nM elacridar (MedChemExpress, catalog no. 58407), or 1 μM Ko134 (Sigma-Aldrich, catalog no. K2144-1MG). The KB1P breast tumor and BCBM tumor organoids were treated with 10 nM doxorubicin (Actavis), 20 nM topotecan (Sandoz), 20 nM paclitaxel (Fresenius Kabi), or 200 nM elacridar (MedChemExpress, catalog no. 58407).

### Cytotoxicity assays

A total of 8,000 cells were seeded in 5 μl of BME in a 96-well plate and treated with 30 nM doxorubicin (Actavis) for 120 hours. Next, cells were incubated with CellTiter-Blue (Promega, catalog no. G8080) for 4 hours in the dark.

### Cleaved caspase 3 immunofluorescent staining, confocal imaging, and analysis

Following treatment, organoids were fixed in 4% paraformaldehyde in PBS for 20 min at room temperature. Organoids were next permeabilized with 0.2% Triton X-100 in PBS for 15 min at room temperature. A blocking step was next performed using 5% bovine serum albumin diluted in PBS for 2 hours at room temperature, followed by staining overnight at 4°C with anti–cleaved caspase 3 (Asp^175^, Cell Signaling Technology, catalog no. 9661; 1:400). Appropriate Alexa Fluor–labeled secondary antibody (Thermo Fisher Scientific) was combined with 4′,6-diamidino-2-phenylindole (DAPI; 1 μg/ml) and incubated for 1 hour in the dark at room temperature. Stained organoids were imaged on an inverted Leica TCS SP8 confocal microscope (Mannheim, Germany), in 8 bit with a 25× water immersion objective (HCX PL APO CS 25.0x0.70 WATER UV). ImageJ was used to quantify the number of cleaved caspase 3–positive cells. The percentage of cleaved caspase 3–positive cells in untreated samples was subtracted to the treated group and plotted in the figures of this manuscript.

### Organoids transduction

Organoids were transduced either with a construct containing H2B-Dendra2-luciferase, luciferase, and shRNA constructs or with the construct to rescue BCRP expression in the shRNA 1 for BCRP. shRNA constructs were provided by the RNAi Consortium mouse library. Resistance cassette from the shRNA BCRP vectors was changed from puromycin into blasticidin. Control shRNA pLKO.1 was purchased from Addgene (plasmid #26701). The rescue construct was produced by VectorBuilder where a Kozak and P2A sequence were included, as well as a hygromycin resistance cassette (table S2). Lentivirus was generated by transient transfection of human embryonic kidney 293FT cells, as described before (*64*). Lentiviral titers were determined using the quantitative polymerase chain reaction (qPCR) Titration Kit (Applied Biological Materials, catalog no. LV900), following the manufacturer’s instructions. For all experiments, the amount of lentiviral supernatant used was calculated to achieve a multiplicity of infection of 5 or 25. Organoids were trypsinized into smaller clusters of approximately eight cells and incubated with the virus, polybrene (100 μg/ml; Sigma, catalog no. TR-1003-G) and 10 μM Y-27632 (Bio Connect, catalog no. S1049) on a 48-well plate (Greiner, catalog no. 677970). Spin infection was done at 600 rcf for 1 hour at 32°C, and organoids were subsequently incubated at 37°C for 6 hours. Next, organoids were washed twice with DMEM/F12 GlutaMAX medium (Gibco, catalog no. 10565018) and plated in BME. Complete DMEM/F12 GlutaMAX medium (Gibco, catalog no. 10565018), supplemented with 10 mM Hepes (Gibco, catalog no. 15630106), streptomycin (100 μg/ml), penicillin (100 U/ml; Gibco, catalog no. 15140122), FGF (12 ng/ml; Gibco, catalog no. PHG0261), 2% B27 supplement (Gibco, catalog no. 17504001), and 10 μM Y-27632 (Bio Connect, catalog no. S1049), was added to the organoids for 2 days. Virus titers were calculated with a qPCR lentivirus titration titer kit (Applied Biological Materials, catalog no. LV900), and a multiplicity of infection of 5 was used for transductions. Organoids transduced with shRNAs were selected with blasticidin (10 μg/ml; Gibco, catalog no. A1113903).

### RNA isolation, cDNA isolation, and real-time qPCR

For RNA extraction of cultured organoids, ~1 × 10^6^ cells were harvested. Cells were lysed in TRIzol (Thermo Fisher Scientific, 15596018), and RNA was extracted using standard TRIzol-chloroform extraction methods. RNA concentration and purity were measured using DS-11/DS-11+ Spectrophotometer (DeNovix). RNA (1000 ng) was used to prepare cDNA using the Reverse Transcription Kit (Applied Biosystems, catalog no. 4368814) according to the manufacturer’s protocol. The generated cDNA was used for real-time qPCR using the SYBR Green Master mix in a QuantStudio Real-Time qPCR system, using the following primers: *BCRP*, 5′-CAGTTCTCAGCAGCTCTTCGAC-3′ (forward) and 5′-TCCTCCAGAGATGCCACGGAT-3′ (reverse); *RPL38*, 5′-AGGATGCCAAGTCTGTCAAGA-3′ (forward) and 5′-TCCTTGTcTGTGATAACCAGGG-3′ (reverse); ABCA6, 5′-CTGAACCTGGAAGGAGAACCAAG-3′ (forward) and 5′-TGGTGCTCACAGTCTCCTGAAC-3′ (reverse); ABCA8, 5′-GCTTTGCCAGAGTCTTGACAGC-3′ (forward) and 5′-TCCTTCTCCCACGATGTCAACC-3′ (reverse); ABCB5, 5′-TGACCACGCAAAGCGAAGAACG-3′ (forward) and 5′-CGCCATAATCCTCAATGCCTTGG-3′ (reverse); ABCC9, 5′-TGAAGCACTCGGTGATTGTGGC-3′ (forward) and 5′-AATGCCTGCTCCACAGAGGATG-3′ (reverse); ABCD2, 5′-CCATAGCAAGCGTGGAGGTAAC-3′ (forward) and 5′-CACTTCGCCCGCTGGTGTAATT-3′ (reverse); ABCA9, 5′-TGACCACGCAAAGCGAAGAACG-3′ (forward) and 5′-CGCCATAATCCTCAATGCCTTGG-3′ (reverse). Expression values were calculated by transforming *C*_t_ values (2^−*C*t^) and were normalized to the mean value of the transformed *C*_t_ values of the reference gene *RPL38*.

### Analysis of BCRP expression in the Cosgrove dataset.

We analyzed the differential expression of genes from the ABC transporter family in BCBM compared to patient-matched primary tumors published by Cosgrove *et al*. (*26*). In Fig. 4, we represented the log_2_ fold change of those genes that are significantly differentially expressed in BCBM compared to patient-matched primary tumors in the Her2^+^ cohort.

### Statistical analysis

The normality of data was tested using the Shapiro-Wilk test. For all normally distributed measurements, one-way analysis of variance (ANOVA) (when >2 means were compared) followed by Tukey’s multiple comparisons test or parametric *t* test was used to determine significance, set to *P* < 0.05. Kaplan-Meier survival curves were analyzed with a log-rank Mantel-Cox test. Tumor growth curve *P* values were calculated using a mixed-effects model with the Geisser-Greenhouse correction. All statistical analyses were performed using GraphPad Prism software (version 9.1.2, GraphPad Software, USA).
