# Supplementary material for: BCRP drives intrinsic chemoresistance in chemotherapy-naïve breast cancer brain metastasis
Source: Sci Adv. 2023 Oct 18;9(42):eabp9530. doi: 10.1126/sciadv.abp9530 (PMC10584345; doi:10.1126/sciadv.abp9530)
Supplement: Supplementary file 1 — Figs. S1 to S6 Table S2 Legend for table S1 [file sciadv.abp9530_sm.pdf]

Science Advances
AAAS

# Supplementary Materials for

## BCRP drives intrinsic chemoresistance in chemotherapy-naïve breast cancer brain metastasis

Rebeca Uceda-Castro *et al.*

Corresponding author: Claire Vennin, c.vennin@nki.nl; Jacco van Rheenen, j.v.rheenen@nki.nl

*Sci. Adv.* **9**, eabp9530 (2023)
DOI: 10.1126/sciadv.abp9530

**The PDF file includes:**

Figs. S1 to S6
Table S2
Legend for table S1

**Other Supplementary Material for this manuscript includes the following:**

Table S1

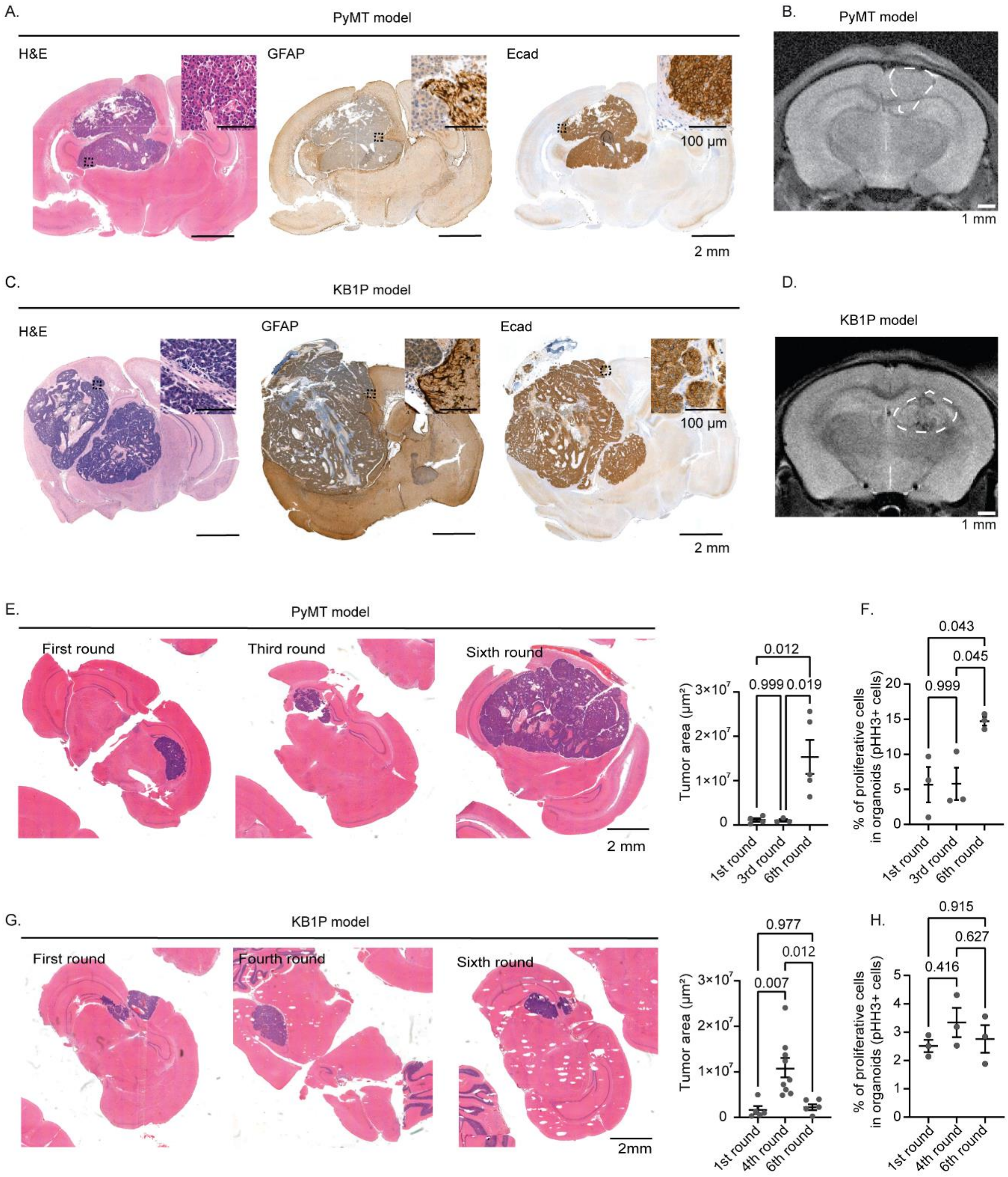

A.
PyMT model
H&E
GFAP
Ecad
100 µm
2 mm
B.
PyMT model
1 mm
C.
KB1P model
H&E
GFAP
Ecad
100 µm
2 mm
D.
KB1P model
1 mm
E.
PyMT model
First round
Third round
Sixth round
2 mm
Tumor area (µm²)
0.012
0.999
0.019
1st round
3rd round
6th round
F.
% of proliferative cells in organoids (pHH3+ cells)
0.043
0.045
0.999
1st round
3rd round
6th round
G.
KB1P model
First round
Fourth round
Sixth round
2mm
Tumor area (µm²)
0.977
0.012
0.007
1st round
4th round
6th round
H.
% of proliferative cells in organoids (pHH3+ cells)
0.915
0.627
0.416
1st round
4th round
6th round

## S1. Characterization of BCBM models

**A.** Representative images of H&E staining and immunohistochemistry staining for GFAP and E-Cadherin PyMT#1 BCBM. **B.** Representative example of Magnetic Resonance Imaging (MRI) of PyMT#1 BCBM in mice. The dotted line marks the BCBM edge. **C.** Representative images of H&E staining and immunohistochemistry staining for GFAP and E-Cadherin in KB1P BCBM. **D.** Representative example of Magnetic Resonance Imaging (MRI) of KB1P BCBM in mice. The dotted line marks the BCBM edge. For A-D, BCBM cells were injected intracranially, tumor formation and localization in the brain parenchyma at humane endpoint was confirmed by H&E. **E.** Representative images of H&E staining and quantification of tumor area at endpoint in brain tumors generated upon intracranial injection of organoids derived from either the mammary tumors (first round), third round or sixth round of brain enrichment and quantification of the tumor area in the PyMT model (n= 5 mice *per* group). **F.** Quantification of cell proliferation using immunofluorescence staining for phospho histone H3 in organoids derived from either the mammary tumors (first round), third round or sixth round of brain enrichment and quantification of the tumor area in the PyMT model. **G.** Representative images of H&E staining and quantification of tumor area at endpoint in brain tumors generated upon intracranial injection of organoids derived from either the mammary tumors (first round), fourth round or sixth round of brain enrichment and quantification of the tumor area in the KB1P model (5 mice *per* group for the first and sixth round and 9 mice for the fourth round). **H.** Quantification of cell proliferation using immunofluorescence staining for phospho histone H3 in organoids derived from either the mammary tumors (first round), fourth round or sixth round of brain enrichment and quantification of the tumor area in the model.

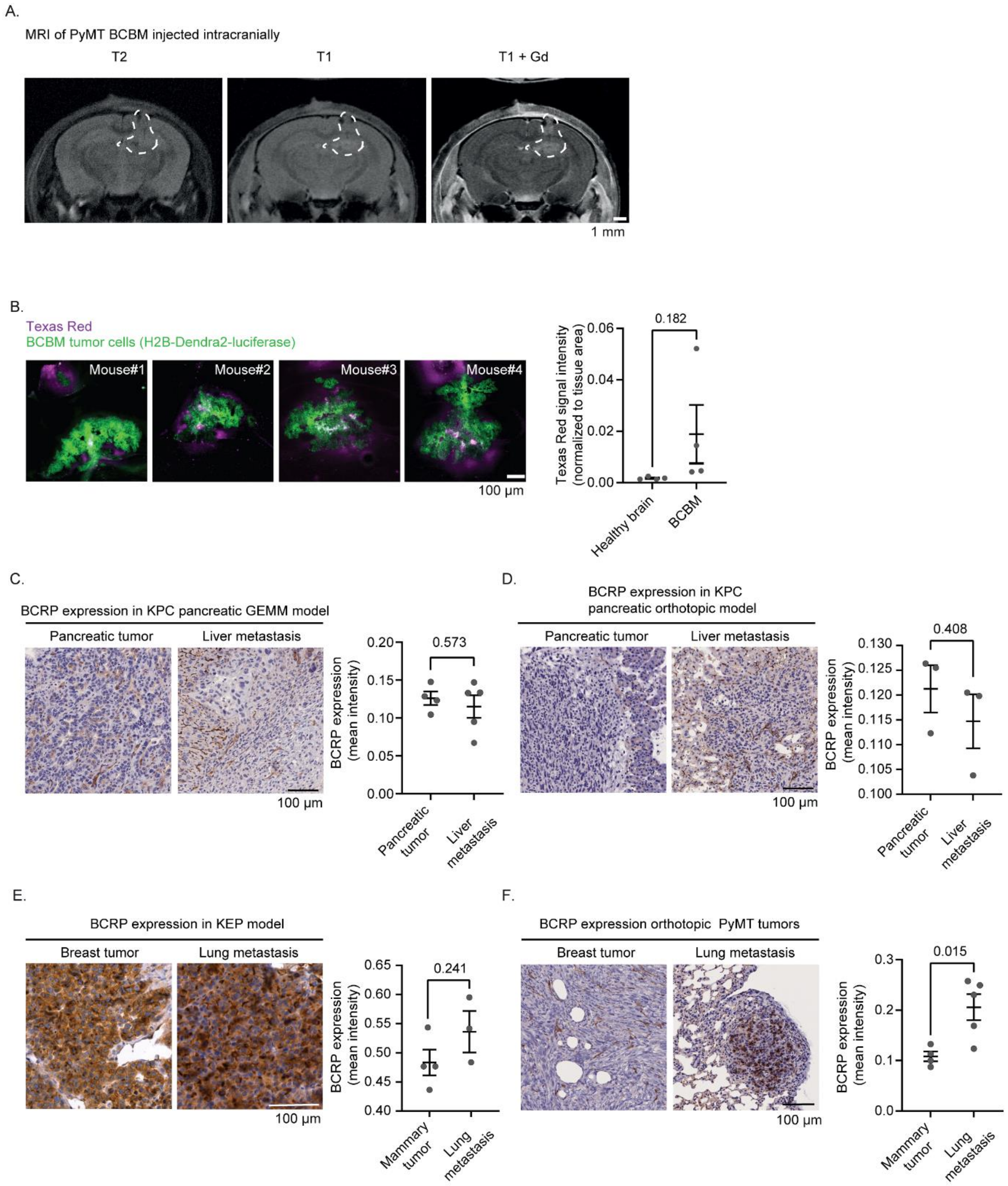


**S2. Characterization of the vasculature of intracranial tumor and BCRP expression in other mouse models of extracranial metastases.**

**A.** Representative example of T2-weighted, T1-weighted pre-contrast and T1-weighted post gadolinium (Gd) contrast Magnetic Resonance Imaging (MRI) of PyMT BCBM, two weeks after

intracranial injection. **B.** Quantification of fluorescence microscopy of Texas Red (purple) detected in the healthy brain and the PyMT BCBM 2 weeks after intracranial injection of PyMT BCBM organoids (green, H2B-Dendra2-luciferase). **C.** Representative images and quantification of BCRP staining in pancreatic tumors and matched liver metastasis from the KPC GEMM model. n=4 pancreatic tumors and 5 liver metastases **D.** Representative images and quantification of BCRP staining in pancreatic tumors and matched liver metastasis in mice orthotopically injected with tumor and CAFs cells derived from KPC tumors. n=3 mice. **E.** Representative images and quantification of BCRP staining in breast tumors and matched lung metastases from k14Cre;Cdh1$^{f/f}$;Trp53$^{f/f}$ (KEP model). n=3 mice. **F.** Representative images and quantification of BCRP staining in breast tumor and matched lung metastasis from PyMT orthotopic model, n=3 mice. Data are presented as mean +/- SEM

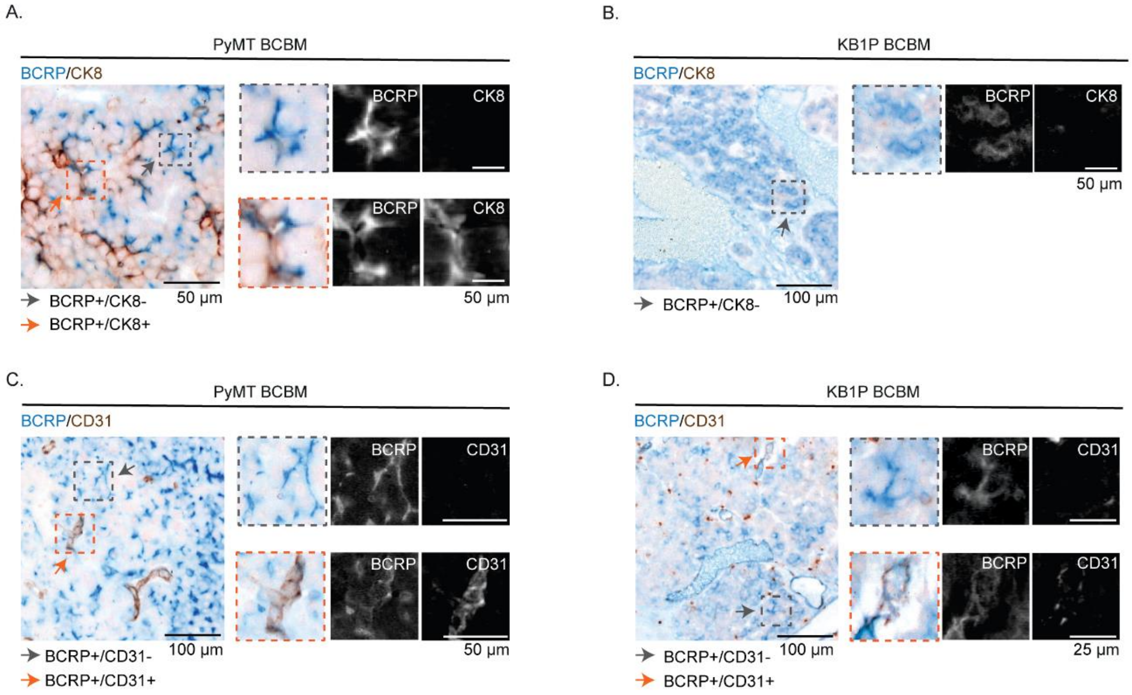


## S3. Method for characterization of vascular mimicry by immunohistochemistry.

**A.** Representative images of immunohistochemistry dual staining of BCRP (blue) and CK8 (brown) in PyMT BCBM. **B.** Representative images of immunohistochemistry dual staining of BCRP (blue) and CK8 (brown) in KB1P BCBM. **C.** Representative images of immunohistochemistry dual staining of BCRP (blue) and CD31 (brown) in PyMT BCBM **D.** Representative images of immunohistochemistry dual staining of BCRP (blue) and CD31 (brown) in KB1P BCBM. Grey arrows indicate single positive staining and orange arrows indicate double positive staining.

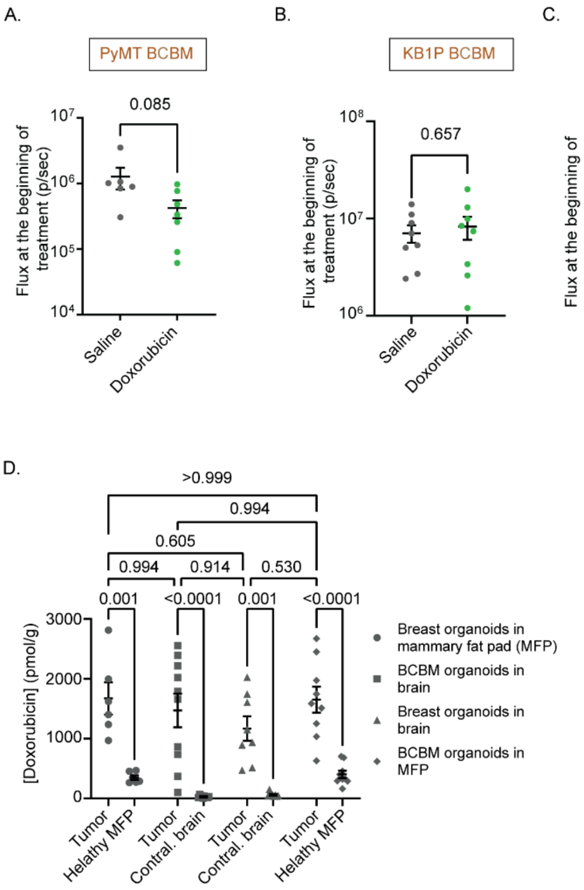


**<u>S4. Control experiment: equal distribution of mice in treatment groups based on IVIS signal and independency of doxorubicin penetration.</u>**

**A. to C.** Quantification of IVIS bioluminescent flux signal at the beginning of the treatment in mice bearing (A.) PyMT BCBM (saline treatment, n=6; doxorubicin, n=7), (B.) KB1P BCBM (n=8 *per* group) and (C) PyMT primary tumor organoids injected intracranially (n=6 mice *per* group). **D.** Quantification of doxorubicin concentration in tumors growing in the mammary fat pad compared to the healthy mammary fat pad (n=6 mice), the BCBM organoids intracranially injected compared to the healthy contralateral hemisphere (n=10 mice), upon injection of primary tumor organoids in the mammary fat pad (n= 9 mice) or BCBM organoids injected in the mammary fat pad (n=9 mice). Please note that these data is compared to data also depicted in Figure 1 E. Data are presented as mean +/- SEM.

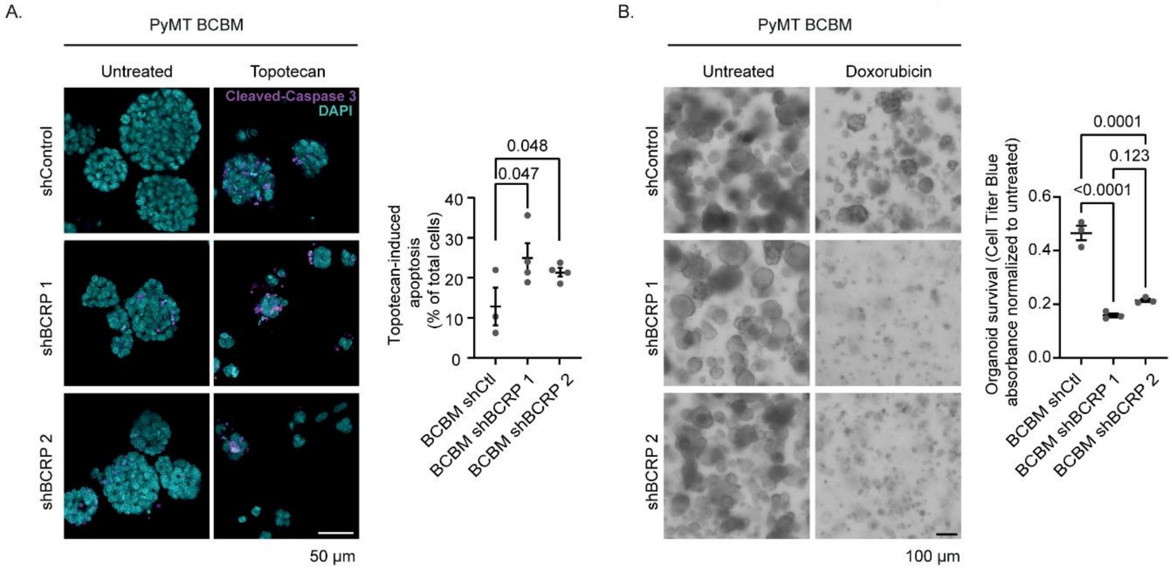


**S5. Reducing BCRP expression sensitizes organoids to doxorubicin and topotecan.**

**A.** Representative images and quantification of cleaved-caspase 3 immunohistochemical staining upon treatment with topotecan and **B.** representative images of organoids at endpoint and quantification of survival using Cell Titer Blue in PyMT BCBM organoids engineered with a shRNA control (shControl) or with shRNA against BCRP (shBCRP 1 and shBCRP 2), and upon treatment with doxorubicin. n=3 biological repeats with 1 technical replicate *per* repeat for cleaved-caspase

3 data, and n=3 biological repeats with 3 technical replicates *per* repeat for Cell Titer blue data. Data are presented as mean +/- SEM.

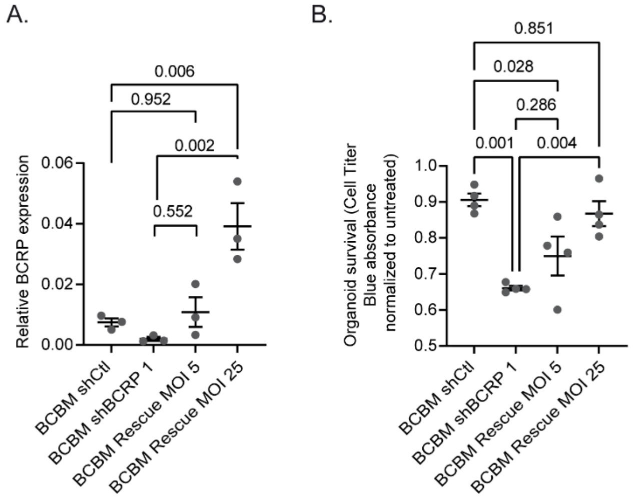


**S6. Rescue of BCRP expression reduces sensitivity in BCBM organoids to doxorubicin.**

**A.** Relative BCRP expression in PyMT#1 organoids engineered with a shRNA control (shControl) or with shRNAs against BCRP (shBCRP 1) and shBCRP 1 transduced with BCRP cDNA , as assessed by RT-qPCR. n=3 biological repeats with 3 technical replicates *per* repeat. **B.** Quantification of survival using Cell Titer Blue in PyMT BCBM organoids engineered with a shRNA control (shControl) or with shRNA against BCRP (shBCRP 1) or BCRP cDNA, and upon treatment with doxorubicin. n=3 biological repeats with 1 technical replicate *per* repeat for cleaved-caspase 3 data, and n=4 biological repeats with 2 technical replicates *per* repeat for Cell Titer blue data. Data are presented as mean +/- SEM.

**Table S1. RNA sequencing of BCBM organoids *versus* primary tumor organoids in the PyMT model.** Genes highlighted in purple are associated with a brain-like phenotype. Positive log2fold change reports genes overexpressed in the BCBM organoids.

**Table S2: Sequence of shRNA and rescue constructs used in the study.**

| Name | Sequence |
|---|---|
| shRNA BCRP 1 | GCCAGTCTATGTTACCTCTTT |
| shRNA BCRP 2 | CCCTGGCTTGTATGATTATTA |
| Rescue BCRP | GCCACCATGTCTTCCAGTAATGACCACGTGTTAGTACCAATGTCGCA<br>GAGAAACAACAACGGCCTTCCTAGGATGAACTCCAGAGCCGTTAGG<br>ACGCTCGCAGAAGGAGATGTGTTGAGTTTTCATCACATCACCTATCG<br>AGTGAAAGTAAAGAGTGGGTTTCTAGTCCGGAAAACAGTTGAGAAA<br>AAATACTATCAGATATCAATGGGATCATGAAACCTGGCCTTAATGCT<br>ATTCTGGGACCCACAGGCGGAGGCAAGTCTTCGTTGCTAGATGTCT<br>TAGCAGCAAGGAAAGATCCAAAGGGATTATCTGGAGATGTTTTGATA<br>AATGGAGCACCTCAACCTGCCCATTTCAAATGCTGTTCAGGTTATGT<br>GGTTCAAGATGACGTTGTGATGGGCACCCTGACAGTGAGAGAAAAC<br>TTACAGTTCTCAGCAGCTCTTCGACTTCCAACAACTATGAAGAATCA<br>GAAAAAAATGAACGGATTAACACAATCATTAAAGAGTTAGGTCTGGA<br>AAAAGTAGCAGATTCTAAGGTCGGAACTCAGTTTACCGTGGCATCTC<br>TGGAGGAGAAAGAAAAAGGACAAGCATAGGGATGGAGCTGATCACT<br>GACCCTTCCATCCTCTTCCTGGATGAGCCCACGACTGGTTTGGACT<br>AAGCACAGCGAATGCTGTCCTTTTGCTCCTGAAAAGGATGTCTAAAC<br>AGGGTCGAACAATCATCTTCTCCATTCATCAGCCTCGGTATTCCATC<br>TTTAAGTTGTTTGACAGCCTCACCTTACTGGCTTCCGGGAAACTCGT<br>GTTCCATGGGCCAGCACAGAAGGCCTTGGAGTACTTTGCATCAGCA<br>GGTTACCACTGTGAGCCCTACAACAACCCTGCGGATTTTTTCCTTGA<br>TGTCATCAATGGAGATTCTTCTGCTGTGATGTTAAATAGAGAGGAAC<br>AAGACAATGAAGCAAACAAGACTGAAGAGCCTTCCAAGGGAGAGAA<br>GCCAGTAATAGAAAATTTATCTGAGTTTTATATCAACTCTGCCATCTA<br>TGGAGAAACAAAAGCTGAATTAGATCAACTTCCAGGAGCTCAGGAA<br>AAGAAAGGAACATCGGCCTTCAAAGAGCGAGTCTTTGTTACCTTTAC<br>TGTCACCAGCTCCGATGGATTGCCAGGCGCTCATTTAAAAACTTGCT<br>CGGGAACCCTCAAGCTTCTGTTGCTCAGTTAATTGTTACAGTCATAC<br>TGGGGCTTATTATTGGTGCCATTTACTTTGATCTGAAATATGATGCC<br>GCTGGAATGCAAAATAGAGCTGGAGTTTTGTTTTTCCTGACTACCAA<br>CCAGTGTTTTTCCAGTGTGTCAGCTGTGGAGCTGTTCGTAGTGAGA<br>AGAAACTCTTCATACATGAGTACATCAGTGGATATTACAGAGTGTCT<br>TCTTACTTCTTTGGAAAGGTGATGTCTGATTTACTCCCCATGAGGTT<br>CTTGCCAAGTGTTATATTCACTTGTATATTATACTTCATGTTAGGACT<br>GAAGAAGACGGTGGATGCTTTTTTCATCATGATGTTTACCCTTATAAT<br>GGTGGCTTATACGGCCAGTTCCATGGCACTGGCCATAGCCACAGGC<br>CAAAGTGTGGTGTCTGTAGCAACACTTCTCATGACAATCGCTTTTGT<br>ATTTATGATGCTCTTTTCTGGCCTCTTGGTGAATCTCAGAACCATTG<br>GGCCTTGGCTGTCCTGGCTTCAGTACTTTAGCATTCCTCGATATGGC<br>TTCACAGCTTTGCAGTATAATGAATTCTTGGGACAAGAGTTTTGTCC<br>AGGATTCAATGTAACGGACAACAGCACTTGTGTTAACAGCTATGCAA<br>TATGTACTGGTAACGAGTACTTGATAAATCAGGGCATCGAACTGTCA<br>CCTTGGGGACTGTGGAAGAATCATGTGGCCCTGGCTTGTATGATTA<br>TTATCTTCCTCACAATTGCCTACCTGAAATTGTTGTTTCTTAAAAAGT |

| | |
|---|---|
| | ATTCTGCCACCAACTTCTCCCTGCTGAAGCAGGCCGGCGACGTGGA<br>GGAGAACCCCGGCCCCAAGCTCCCGGGAGCTTGTATATCCATTTTC<br>GGATCTGATCAGCACGTGATGAAAAAGCCTGAACTCACCGCGACGT<br>CTGTCGAGAAGTTTCTGATCGAAAAGTTCGACAGCGTCTCCGACCT<br>GATGCAGCTCTCGGAGGGCGAAGAATCTCGTGCTTTCAGCTTCGAT<br>GTAGGAGGGCGTGGATATGTCCTGCGGGTAAATAGCTGCGCCGAT<br>GGTTTCTACAAAGATCGTTATGTTTATCGGCACTTTGCATCGGCCGC<br>GCTCCCGATTCCGGAAGTGCTTGACATTGGGGAATTCAGCGAGAGC<br>CTGACCTATTGCATCTCCCGCCGTGCACAGGGTGTCACGTTGCAAG<br>ACCTGCCTGAAACCGAACTGCCCGCTGTTCTGCAGCCGGTCGCGG<br>AGGCCATGGATGCGATCGCTGCGGCCGATCTTAGCCAGACGAGCG<br>GGTTCGGCCCATTCGGACCGCAAGGAATCGGTCAATACACTACATG<br>GCGTGATTTCATATGCGCGATTGCTGATCCCCATGTGTTCACTGGCA<br>AACTGTGATGGACGACACCGTCAGTGCGTCCGTCGCGCAGGCTCT<br>CGATGAGCTGATGCTTTGGGCCGAGGACTGCCCCGAAGTCCGGCA<br>CCTCGTGCACGCGGATTTCGGCTCCAACAATGTCCTGACGGACAAT<br>GGCCGCATAACAGCGGTCATTGACTGGAGCGAGGCGATGTTCGGG<br>GATTCCCAATACGAGGTCGCCAACATCTTCTTCTGGAGGCCGTGGT<br>TGGCTTGTATGGAGCAGCAGACGCGCTACTTCGAGCGGAGGCATC<br>CGGAGCTTGCAGGATCGCCGCGGCTCCGGGCGTATATGCTCCGCA<br>TTGGTCTTGACCAACTCTATCAGAGCTTGGTTGACGGCAATTTCGAT<br>GATGCAGCTTGGGCGCAGGGTCGATGCGACGCAATCGTCCGATCC<br>GGAGCCGGGACTGTCGGGCGTACACAAATCGCCCGCAGAAGCGCG<br>GCCGTCTGGACCGATGGCTGTGTAGAAGTACTCGCCGATAGTGGAA<br>ACCGACGCCCCAGCACTCGTCCGAGGGCAAAGGAATAG |
